# Comparative Effectiveness of 2 Interventions to Increase Breast, Cervical, and Colorectal Cancer Screening Among Women in the Rural US: A Randomized Clinical Trial

**DOI:** 10.1001/jamanetworkopen.2023.11004

**Published:** 2023-04-28

**Authors:** Victoria L. Champion, Electra D. Paskett, Timothy E. Stump, Erika B. Biederman, Eric Vachon, Mira L. Katz, Susan M. Rawl, Ryan D. Baltic, Carla D. Kettler, Eric E. Seiber, Wendy Y. Xu, Patrick O. Monahan

**Affiliations:** 1 School of Nursing, Indiana University, Indianapolis; 2 Indiana University Melvin and Bren Simon Comprehensive Cancer Center, Indianapolis; 3 Comprehensive Cancer Center, The Ohio State University, Columbus; 4 Division of Cancer Prevention and Control, Department of Medicine, College of Medicine, The Ohio State University, Columbus; 5 Department of Biostatistics and Health Data Science, Indiana University School of Medicine, Indianapolis; 6 Center for Health Services Research, Regenstrief Institute, Indianapolis, Indiana; 7 Division of Health Behavior and Health Promotion, College of Public Health, The Ohio State University, Columbus; 8 Division of Health Services Management and Policy, The Ohio State University, Columbus

## Abstract

**Importance:**

Women living in rural areas have lower rates of breast, cervical, and colorectal cancer screening compared with women living in urban settings.

**Objective:**

To assess the comparative effectiveness of (1) a mailed, tailored digital video disc (DVD) intervention; (2) a DVD intervention plus telephonic patient navigation (DVD/PN); and (3) usual care with simultaneously increased adherence to any breast, cervical, and colorectal cancer screening that was not up to date at baseline and to assess cost-effectiveness.

**Design, Setting, and Participants:**

This randomized clinical trial recruited and followed up women from rural Indiana and Ohio (community based) who were not up to date on any or all recommended cancer screenings. Participants were randomly assigned between November 28, 2016, and July 1, 2019, to 1 of 3 study groups (DVD, DVD/PN, or usual care). Statistical analyses were completed between August and December 2021 and between March and September 2022.

**Intervention:**

The DVD interactively assessed and provided messages for health beliefs, including risk of developing the targeted cancers and barriers, benefits, and self-efficacy for obtaining the needed screenings. Patient navigators counseled women on barriers to obtaining screenings. The intervention simultaneously supported obtaining screening for all or any tests outside of guidelines at baseline.

**Main Outcomes and Measures:**

Receipt of any or all needed cancer screenings from baseline through 12 months, including breast, cervical, and colorectal cancer, and cost-effectiveness of the intervention. Binary logistic regression was used to compare the randomized groups on being up to date for all and any screenings at 12 months.

**Results:**

The sample included 963 women aged 50 to 74 years (mean [SD] age, 58.6 [6.3] years). The DVD group had nearly twice the odds of those in the usual care group of obtaining all needed screenings (odds ratio [OR], 1.84; 95% CI, 1.02-3.43; *P* = .048), and the odds were nearly 6 times greater for DVD/PN vs usual care (OR, 5.69; 95% CI, 3.24-10.5; *P* < .001). The DVD/PN intervention (but not DVD alone) was significantly more effective than usual care (OR, 4.01; 95% CI, 2.60-6.28; *P* < .001) for promoting at least 1 (ie, any) of the needed screenings at 12 months. Cost-effectiveness per woman who was up to date was $14 462 in the DVD group and $10 638 in the DVD/PN group.

**Conclusions and Relevance:**

In this randomized clinical trial of rural women who were not up to date with at least 1 of the recommended cancer screenings (breast, cervical, or colorectal), an intervention designed to simultaneously increase adherence to any or all of the 3 cancer screening tests was more effective than usual care, available at relatively modest costs, and able to be remotely delivered, demonstrating great potential for implementing an evidence-based intervention in remote areas of the midwestern US.

**Trial Registration:**

ClinicalTrials.gov Identifier: NCT02795104

## Key Points

**Question:**

What is the comparative effectiveness of 2 tailored interventions delivered remotely compared with usual care for increasing any or all needed cancer screenings in rural women?

**Findings:**

In this randomized clinical trial of 963 women, digital video disc (DVD) plus patient navigation, compared with usual care, significantly increased the odds of obtaining any needed screening after adjusting for other prognostic covariates.

**Meaning:**

These findings suggest that tailored interventions delivered remotely may increase needed screenings in rural women, ultimately decreasing cancer mortality.

## Teaser

This randomized clinical trial assesses whether 2 tailored interventions delivered remotely were effective at increasing the rate of any or all needed cancer screenings in rural women.

## Introduction

Adherence to guideline-based screening for breast, cervical, and colorectal cancer decreases mortality; unfortunately, rural screening rates fall short of Healthy People 2030 goals.^1^ For instance, compared with residents of large metropolitan areas, people living in rural sectors with fewer than 10 000 residents experience a 12-point higher crude cancer mortality rate.^2^ Previous studies have identified sociodemographic factors that limit up-to-date screening for these cancers in rural areas, including lower educational attainment, less knowledge about screening, lower income, poor access to health care, and greater social deprivation,^3^ as measured by the area deprivation index (ADI).^4,5,6,7,8,9,10^ Given the substantial contribution guideline-based cancer screening provides for lowering cancer mortality,^11,12,13^ interventions to increase breast, cervical, and colorectal cancer screening could increase adherence rates of being up to date with screening guidelines and decrease the disparate cancer mortality experienced by rural women, resulting in cost savings by preventing or finding and treating cancers at earlier stages.^14^

Over the past 2 decades, interventions to improve screening have demonstrated efficacy for tailored messaging delivered through print, telephone, and technology.^14,15,16,17,18^ Furthermore, patient navigation is effective in increasing cancer screening.^19,20,21,22^ Technological advancements with dissemination have allowed both tailored interventions and patient navigation to be delivered remotely via technology or telephone, opening the possibility of reaching rural US residents.^20,21,22^ Although studies have intervened simultaneously to increase the uptake of 2 needed cancer screening tests,^15,16,19,23^ most interventions have focused on screening for a single cancer: breast, cervical, or colorectal. Supporting a multiscreening approach, the literature provides evidence that individuals who complete 1 cancer screening behavior are more likely to complete a second, and experts are now voicing the possibility of providing a “one-stop shop” approach to cancer screening to increase multiple screening rates.^15,16,17,18,19,23^

To our knowledge, no interventions have been tested to simultaneously increase the guideline-recommended breast, cervical, and colorectal cancer screenings for women. Each of these screenings can detect early-stage disease, protecting women from breast, cervical, or colorectal cancer mortality, and addressing all 3 cancers simultaneously increases the probability that women will have knowledge of and consider all screenings for which they are not up to date.

This study evaluated the effect of 2 interventions: (1) a mailed, interactive digital video disc (DVD) with messages tailored to each woman’s responses and (2) the DVD followed by telephonic patient navigation (DVD/PN). Both interventions were tailored to the unique barriers, needs, and experiences of rural women by using platforms that could be delivered remotely, thereby reducing access barriers. Intervention groups were compared with usual care for increasing the percentage of women who were up to date with all recommended screening tests (breast, cervical, or colorectal). Secondary research questions tested the comparative effectiveness of the 2 interventions vs usual care for increasing the percentage of women up to date with screening for any needed screening (breast, cervical, and colorectal cancer). In addition, we assessed the costs and cost-effectiveness of these interventions.

## Methods

### Sample

In this randomized clinical trial, participants were recruited between October 20, 2016 (first baseline interview), and March 15, 2019 (last baseline interview), from 98 rural Indiana and Ohio counties with Rural-Urban Continuum Codes ranging from 4 (least rural) to 9 (most rural).^24^ Eligibility included (1) biological female sex, (2) age 50 to 74 years, (3) not up to date with screening for 1 or more guideline-based cancer screening for women (breast, cervical, or colorectal), (4) ability to speak English, (5) no previous cancer diagnosis (other than nonmelanoma skin cancer), and (6) provision of informed consent. Definitions for being up to date with screening for these cancers were obtained from the US Preventive Task Force (USPSTF)^25,26,27^ and included (1) biennial screening mammography for women aged 50 to 74 years; (2) cervical cytology completed every 3 years or Papanicolaou and human papillomavirus test or cotesting completed every 5 years for women aged 21 to 65 years; and (3) colorectal cancer screening (fecal occult blood test/fecal immunochemical test [annual], colonoscopy [10 years]).^28,29^ Screening verification via medical record review (MRR) was used to both assess baseline eligibility and determine outcomes. The study was approved by the institutional review boards of Indiana University and The Ohio State University, and all participants provided written informed consent. The full trial protocol and statistical analysis plan are available in Supplement 1. This study follows the Consolidated Standards of Reporting Trials (CONSORT) reporting guideline.

Recruitment methods included 3 strategies: (1) commercial listing of women meeting age criteria residing in rural counties in Indiana and Ohio, (2) personal contact at community events, and (3) social media and advertisement websites. During an initial phone call, potentially eligible participants (N = 1852) verbally consented to participate in the study. Participants completed a baseline survey and consented to MRR to verify screening status for each of the 3 cancers. Of the 1852 women, 209 refused to consent to MRR, and 658 were ineligible after MRR, leaving 983 eligible women randomly assigned to study groups (Figure).

**Figure.**
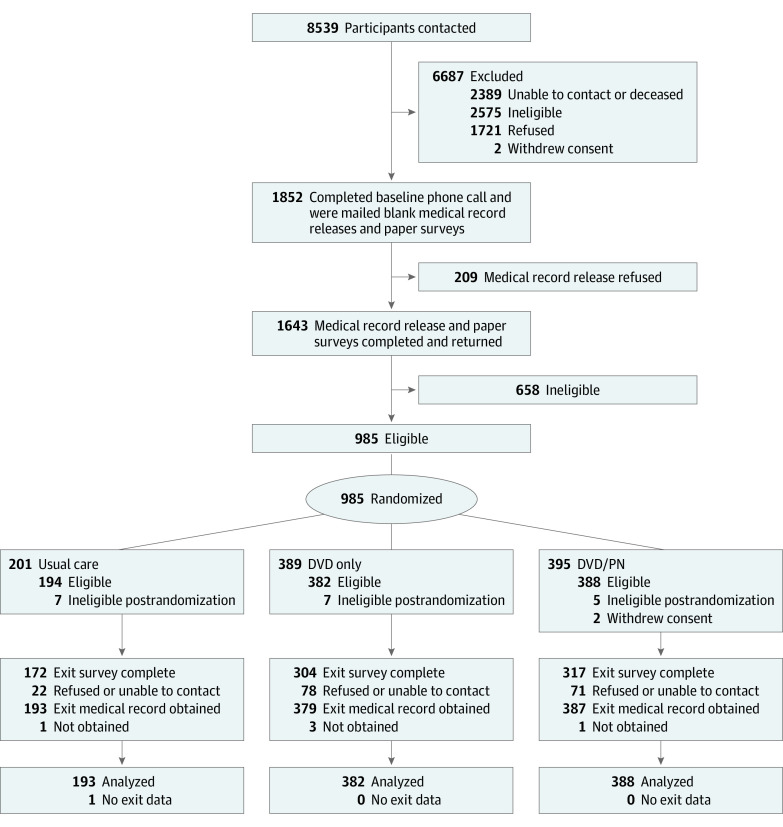
Participant Flow Diagram DVD indicates digital video disc; PN, patient navigation.

### Interventions

An interactive DVD, developed by study investigators (V.L.C. and S.M.R.), allowed users the ability to respond to prompts and receive personalized feedback to encourage uptake of needed screenings. Within each cancer screening unit, tailored messages, built on 2 decades of research,^15,30^ provided information specific to the woman’s age, family history of cancer, perceived risk of developing the specific cancers, and barriers, benefits, and self-efficacy with regard to the respective screening behavior.^15,30,31,32,33,34,35,36,37,38,39^ An explanation of the screening options within each unit included information about the process for scheduling and completing needed screenings.

Participants were randomly assigned to the DVD, DVD/PN, and usual groups between November 26, 2018, and July 1, 2019. Women randomly assigned to the DVD/PN intervention group were mailed a DVD followed by a patient navigator telephone attempt within 4 weeks. Two licensed social workers who were residents of rural Ohio were trained as patient navigators by study investigators (E.D.P. and M.L.K.). Social workers were selected as navigators because they had the requisite knowledge and skills needed to counsel women regarding cancer screening. Navigators contacted participants, confirmed receipt of the DVD, promoted and fostered information provided by the DVD, and counseled women to overcome identified barriers to any screening tests that were needed. Additional follow-up calls were made, as necessary, with a mean of 3 content calls (range, 1-14 calls) successfully completed per woman. A trained research assistant assessed 10% of the calls for fidelity.

### Outcome Assessment

Outcome data (completion of screening tests) were obtained 12 months after mailing the DVD through MRR verification and self-report. Prior to MRR, women were queried about the medical record home(s) of all screening test results. Women were considered up to date at 12 months for all screening tests if breast, cervical, and colorectal cancer screening had been completed consistent with USPSTF guidelines^25,26,27^ during the period between baseline and 12 months for the tests for which they were not up to date at baseline. To become up to date for all needed cancer screening tests, women needed to screen for 1, 2, or 3 cancers depending on baseline status. To become up to date for any needed cancer screening tests, women needed to be screened for at least 1 of the cancer screening tests that were not up to date at baseline. Self-report measures (baseline and 12 months) included sociodemographic and health care variables, smoking status, knowledge about the cancers and their guideline-recommended screening tests, health beliefs, and intention to obtain any screenings that were not up to date. Sociodemographic variables collected included age, education, income, marital status, insurance status, race and ethnicity, employment status, and height and weight to calculate body mass index (weight in kilograms divided by height in meters squared). Race and ethnicity were self-reported and can be an important factor in screening uptake. Physician-related variables contained questions about recommendations for cancer screening tests received or reminders sent from health care facilities. Knowledge and health beliefs for each cancer screening behavior were assessed using Likert response options.^40,41,42,43,44,45^

### Statistical Analysis

Logistic regression was the primary analysis method; all tests were conducted at the *P* < .05 significance level, except *P* < .25 was used to select the initial variable pool for backward removal models. The statistical analysis was performed between August and December 2021 and again between March and September 2022 using R, version 4.2.3 software (R Foundation for Statistical Computing).^46^ Sample size was determined by projected 12-month effect sizes for usual care, DVD, and DVD/PN, estimated respectively at 10%, 20%, and 30% for being up to date for screening for all 3 cancers (primary outcome) and 25%, 35%, and 45% for being up to date for any cancer screening (secondary outcome). To achieve 80% power for logistic regression analyses, including 2-sided tests when comparing 2 intervention groups and 1-sided tests for comparisons with usual care, we planned for an analyzable (ie, at 12 months) sample of 200 in the usual care group and 376 in each of the 2 intervention groups. A power of 80% was realized for the observed 12-month analyzable sample (193 usual care, 379 DVD, and 387 DVD/PN) for all pairwise tests between arms as well as the omnibus test for both outcomes.

Baseline characteristics were descriptively reported for the overall sample and separately for women in each of the 3 groups.^47^ The intention-to-treat approach was used. Binary logistic regression was used to compare the randomized groups on being up to date for all or any cancer screening(s) at 12 months. Baseline variables with *P* < .25 for associations with outcomes (eTable 1 in Supplement 2) were entered into the initial step of a multivariable backward removal logistic regression procedure to compare study groups on primary and secondary outcomes while adjusting for potentially confounding covariates, where the final model was selected based on the lowest (ie, best) Akaike information criterion.^48^ Study group, age, and baseline screening status for each cancer was forced into all models.

A sensitivity analysis considered women 66 years or older as up to date at baseline (and therefore no screening was needed at 12 months) with cervical cancer screening as supported by guidelines^25,26,27^ (eTables 2 and 3 in Supplement 2). We conducted a cost analysis to calculate the cost per additional woman up to date for all needed screenings by accounting for development and intervention costs for the DVD-only and DVD/PN arms separately, excluding any costs purely attributable to research, converted to 2022 US dollars.^49^

## Results

Based on 12-month MRR, 19 women were excluded because updated MRRs indicated they were up to date with all cancer screenings at baseline, 5 participants were missing MRRs but had self-reported screening outcomes that were used in lieu of medical records as done in previous studies,^15^ and 1 participant did not have MRR or self-report and was considered missing, yielding a sample of 963 participants for analyses (Figure). Participants reported a mean (SD) age of 58.6 (6.3) years; 150 (16%) had a high school education or less, 367 (38%) had some college, and 446 (46%) had a college education or higher. Most participants self-reported as White (936 [97%] vs 27 [3%] other race and ethnicity [ie, African American, Asian, Native American, multiple race and ethnicity]), and 743 (77%) were married. Only 179 participants (19%) reported an annual household income less than $40 000, while 351 (36%) had incomes of $40 000 to $79 999, and 396 (41%) disclosed incomes of $80 000 or more. Only 49 participants (5%) reported not having health insurance (Table 1). Baseline data revealed minimal missing data except for weight, which was unknown for 318 participants (33%). Participants were classified into 7 categories according to their up-to-date status for breast, cervical, and colorectal cancer screenings at baseline, with 186 (19%) reporting not being up to date for all 3 tests (Table 1).

**Table 1.** Baseline Characteristics by Study Group

Characteristic	No. (%)^a^				
	Overall (N = 963)	Usual care (n = 193)	DVD (n = 382)	DVD/PN (n = 388)	
Age at consent, mean (SD), y	58.6 (6.3)	58.8 (6.3)	58.2 (6.4)	58.8 (6.3)	
State					
Indiana	378 (39)	70 (36)	159 (42)	149 (38)	
Ohio	585 (61)	123 (64)	223 (58)	239 (62)	
Education					
High school or GED or less	150 (16)	36 (19)	59 (15)	55 (14)	
Some college or associate’s degree	367 (38)	70 (36)	146 (38)	151 (39)	
Bachelor’s degree	250 (26)	58 (30)	99 (26)	93 (24)	
Master’s degree or greater	196 (20)	29 (15)	78 (20)	89 (23)	
Income, $					
<40 000	179 (19)	35 (18)	77 (20)	67 (17)	
40 000-79 999	351 (36)	70 (36)	129 (34)	152 (39)	
≥80 000	396 (41)	78 (40)	165 (43)	153 (39)	
Unknown	37 (4)	10 (5)	11 (3)	16 (4)	
Marital status					
Married or living as married	743 (77)	151 (79)	304 (80)	288 (74)	
Divorced, widowed, or separated	184 (19)	38 (20)	62 (16)	84 (22)	
Never married	34 (4)	3 (2)	15 (4)	16 (4)	
Insurance coverage					
No insurance	49 (5)	12 (6)	22 (5.8)	15 (3.9)	
Public only	85 (9)	11 (6)	36 (9.4)	38 (9.8)	
Private only	669 (70)	140 (73)	262 (69)	267 (69)	
Public and private	159 (17)	30 (16)	61 (16)	68 (18)	
Race and ethnicity					
White	936 (97)	185 (96)	376 (98)	375 (97)	
Other^b^	27 (2.8)	8 (4.1)	6 (1.6)	13 (3.4)
US-based percentile of block group ADI score, mean (SD)	66.5 (16.0)	66.2 (16.0)	66.5 (16.3)	66.8 (15.6)	
Secondary RUCA code (recoded, categorization B), 2 categories					
Urban and large rural city or town	621 (64)	131 (68)	251 (66)	239 (62)	
Small and isolated small rural town	342 (36)	62 (32)	131 (34)	149 (38)	
US-based quintile of Yost index					
First (lowest SES)	149 (17)	27 (15)	54 (16)	68 (19)	
Second	335 (38)	69 (39)	128 (37)	138 (39)	
Third	299 (34)	58 (32)	120 (35)	121 (34)	
Fourth	94 (11)	25 (14)	39 (11)	30 (8.4)	
Fifth (highest SES)	1 (<1)	0	1 (<1)	0	
Currently working for pay					
No	314 (33)	59 (31)	129 (34)	126 (32)	
Yes, part time	186 (19)	52 (27)	63 (16)	71 (18)	
Yes, full time	463 (48)	82 (42)	190 (50)	191 (49)	
Baseline BMI categories					
Normal (<18.5)	126 (13)	32 (17)	51 (13)	43 (11)	
Overweight (18.5 to <25)	191 (20)	38 (20)	74 (19)	79 (20)	
Obese (25 to <30)	328 (34)	69 (36)	132 (35)	127 (33)	
Unknown (≥30)	318 (33)	54 (28)	125 (33)	139 (36)	
Received any reminders to have a mammogram, FOBT, colonoscopy, or Papanicolaou test					
No	353 (37)	63 (33)	151 (40)	139 (36)	
Yes	610 (63)	130 (67)	231 (60)	249 (64)	
Planning to have a mammogram, FOBT, colonoscopy, or Papanicolaou test in the next 6 mo					
No	391 (41)	73 (38)	151 (40)	167 (43)	
Yes	572 (59)	120 (62)	231 (60)	221 (57)	
Perceived barriers first principal component, mean (SD)^c^	0.00 (1.00)	−0.01 (0.91)	−0.06 (1.03)	0.06 (1.01)	
Perceived benefit of screening score (range, 3-15), mean (SD)	11.46 (2.61)	11.65 (2.14)	11.62 (2.68)	11.20 (2.73)	
Perceived cancer risk score (range, 3-9), mean (SD)	5.43 (1.22)	5.48 (1.14)	5.43 (1.19)	5.41 (1.29)	
Perceived cancer screening self-efficacy score (range, 4-20), mean (SD)	17.68 (2.84)	17.69 (3.08)	17.84 (2.61)	17.53 (2.94)	
Knowledge score first principal component, mean (SD)^c^	0.00 (1.00)	−0.03 (1.01)	0.06 (0.97)	−0.04 (1.02)	
Not up to date for cancer screening at baseline					
Breast, colorectal, and cervical	186 (19)	37 (19)	75 (20)	74 (19)	
Breast and colorectal	89 (9)	18 (9)	34 (9)	37 (10)	
Breast and cervical	68 (7)	15 (8)	26 (7)	27 (7)	
Colorectal and cervical	126 (13)	25 (13)	49 (13)	52 (13)	
Breast only	59 (6)	13 (7)	22 (68)	24 (6)	
Colorectal only	262 (27)	50 (26)	108 (28)	104 (27)	
Cervical only	173 (18)	35 (18)	68 (18)	70 (18)	

Abbreviations: ADI, area deprivation index; BMI, body mass index as calculated by weight in kilograms divided by height in meters squared; DVD, digital video disc; FOBT, fecal occult blood test; GED, General Educational Development test; PN, patient navigation; RUCA, rural-urban commuting area; SES, socioeconomic status.

^a^ Some columns do not total to 100% due to missing data. Total number of missing values was 56 for ADI and 85 for Yost index and ranged from 8 to 10 for scale scores.

^b^ Other included African American, Asian, Native American, and multiple or other race and ethnicity. These categories were grouped together due to the low numbers of participants.

^c^ For purposes of this trial, which investigated combined screening outcomes (all and any up to date), the barrier scores and knowledge scores for each screening test were each combined into a single variable using the first principal component from a principal components analysis.

### Descriptive and Bivariate Analyses

The unadjusted 12-month rate of being up to date with screening for all cancers was 10%, 15%, and 30%, respectively, for usual care, DVD alone, and DVD/PN (omnibus *P* < .001) (Table 2). The unadjusted 12-month rate of being up to date with screening for any of the 3 cancers needed was 25%, 29%, and 49%, respectively (omnibus *P* < .001) (Table 2). The DVD/PN group demonstrated a significantly greater percentage (vs DVD alone or usual care) of women being up to date for all and any needed screenings by 12 months (*P* < .001 for 4 pairwise comparisons).

**Table 2.** Bivariate Analysis of 12-Month Medical Record Cancer Screening Outcomes by Study Group

Characteristic	No. (%)				*P* value^a^			
	Overall (N = 963)	Usual care (n = 193)	DVD (n = 382)	DVD/PN (n = 388)	Omnibus	DVD vs usual care	DVD/PN vs usual care	DVD/PN vs DVD
**Up to date for all cancer screenings within 12 mo of enrollment**								
No record of test or outside window	769 (80)	174 (90)	325 (85)	270 (70)	<.001	.09	<.001	<.001
Received within 12-mo window	194 (20)	19 (10)	57 (15)	118 (30)				
**Up to date for any (ie, at least 1) cancer screening within 12 mo of enrollment**								
No record of test or outside window	616 (64)	145 (75)	272 (71)	199 (51)	<.001	.32	<.001	<.001
Received within 12-mo window	347 (36)	48 (25)	110 (29)	189 (49)				

Abbreviations: DVD, digital video disc; PN, patient navigation.

^a^ Pearson χ^2^ test of 2-sided alternative hypothesis.

### Comparative Effectiveness Analyses for Up-to-Date Screening for All Cancers

After adjusting for a parsimonious set of covariates through backward model selection, women assigned to the DVD group had nearly twice the odds of those in the usual care group of being up to date for all screenings (odds ratio [OR], 1.84; 95% CI, 1.02-3.43; *P* = .048) (Table 3). Women in the combined DVD/PN group were nearly 6 times more likely to be up to date for all cancer screenings compared with usual care (OR, 5.69; 95% CI, 3.24-10.50; *P* < .001). Women in the DVD/PN group were 3 times more likely to obtain all needed screenings compared with those in the DVD group (OR, 3.09; 95% CI, 2.05-4.68; *P* < .001).

**Table 3.** Logistic Regression Model of Odds of Being Up to Date for All Cancer Screenings Within 12 Months Postbaseline (n = 899)^a^

Characteristic	OR (95% CI)	*P* value
Study group^b^		
Usual care	[Reference]	
DVD	1.84 (1.02-3.43)	.048
DVD/PN	5.69 (3.24-10.5)	<.001
Baseline screening status, not up to date		
Breast, colorectal, and cervical	[Reference]	
Breast and colorectal	2.21 (0.90-5.51)	.08
Breast and cervical	2.71 (1.03-7.06)	.04
Colorectal and cervical	1.08 (0.41-2.77)	.88
Breast only	19.10 (8.18-47.30)	<.001
Colorectal only	5.66 (2.91-12.00)	<.001
Cervical only	2.83 (1.35-6.33)	.008
Age, y		
50-54	[Reference]	
55-59	1.00 (0.61-1.63)	>.99
60-64	0.89 (0.52-1.48)	.65
≥65	0.53 (0.30-0.93)	.03
Planning to have a mammogram, FOBT, colonoscopy, or Papanicolaou test in the next 6 mo		
No	[Reference]	
Yes	1.86 (1.24-2.81)	.003
Perceived cancer screening self-efficacy score (range, 4-20)	1.10 (1.01-1.19)	.03
National percentile of block group ADI score	0.99 (0.97-0.998)	.02

Abbreviations: ADI, area deprivation index; DVD, digital video disc; FOBT, fecal occult blood test; OR, odds ratio; PN, patient navigation.

^a^ Reconfirmation of medical record location at 12 months was also adjusted for in this model (confirmed vs not confirmed at 12 months: OR, 4.76; 95% CI, 2.22-12.50; *P* < .001). For the 12% of participants whose medical record health care system location was not confirmed at 12 months, the location reported at their baseline interview was used to assess 12-month outcomes. Only 5 participants had no medical record data or location confirmation at baseline or 12 months, among 4 of whom the 12-month self-report data were available and used in all analyses.

^b^ Effect of DVD/PN vs DVD intervention: OR, 3.09; 95% CI, 2.05-4.68; *P* < .001.

Baseline screening status was significantly associated with 12-month screening up-to-date status. Compared with women not up to date with all screenings at baseline, those who were not up to date for 1 cancer screening or not up to date for 2 cancer screenings, 1 of which included breast cancer screening, were more likely to be up to date for all needed cancer screenings at 12 months (OR, 19.10; 95% CI, 8.18-47.30; *P* < .001) (Table 3). Participants aged 65 years or older were less likely to be up to date for all cancer screenings (OR, 0.53; 95% CI, 0.30-0.93; *P* = .03). Participants who were planning at baseline to obtain cancer screening in the next 6 months (OR, 1.86; 95% CI, 1.24-2.81; *P* = .003), those with higher baseline self-efficacy scores (OR, 1.10; 95% CI, 1.01-1.19; *P* = .03), and those with lower ADI scores (OR, 0.99; 95% CI, 0.97-0.998; *P* = .02) were more likely to be up to date for screening for all cancers at 12 months.

### Comparative Effectiveness Analysis for Being Up to Date for Screening for Any Cancer

In the covariate-adjusted model, the DVD/PN intervention, but not the DVD intervention alone, was significantly more effective than usual care (OR, 4.01; 95% CI, 2.60-6.28; *P* < .001) for promoting an up-to-date screening status for any of the cancers at 12 months (Table 4). The DVD/PN intervention compared with the DVD alone was significantly more effective for promoting up-to-date screening at 12 months (OR, 2.98; 95% CI, 2.09-4.18; *P* < .001).

**Table 4.** Logistic Regression Model of Odds of Being Up to Date for Any (at Least 1) Cancer Screening Within 12 Months Postbaseline (n = 891)^a^

Characteristic	OR (95% CI)	*P* value
Study group^b^		
Usual care	1 [Reference]	
DVD	1.36 (0.88-2.12)	.17
DVD/PN	4.01 (2.60-6.28)	<.001
Baseline screening status, not up to date		
Breast, colorectal, and cervical	1 [Reference]	
Breast and colorectal	0.96 (0.52-1.73)	.89
Breast and cervical	1.35 (0.71-2.56)	.36
Colorectal and cervical	0.80 (0.47-1.36)	.40
Breast only	1.50 (0.75-3.04)	.25
Colorectal only	0.43 (0.27-0.69)	<.001
Cervical only	0.24 (0.13-0.42)	<.001
Age, y		
50-54	1 [Reference]	
55-59	1.16 (0.77-1.75)	.47
60-64	1.06 (0.67-1.67)	.80
≥65	0.91 (0.55-1.49)	.70
Current household financial situation		
Has enough money for special things	1 [Reference]	
Can pay bills, but little extra money	0.85 (0.59-1.22)	.38
Has to cut back or has difficulty paying bills	0.45 (0.24-0.81)	.01
Currently working for pay		
No	1 [Reference]	
Yes, part time	1.41 (0.90-2.22)	.14
Yes, full time	1.58 (1.07-2.36)	.02
Planning to have a mammogram, FOBT, colonoscopy, or Papanicolaou test in the next 6 mo		
No	1 [Reference]	
Yes	1.85 (1.33-2.59)	<.001
Perceived barriers first principal component	1.23 (1.03-1.47)	.02
Knowledge score first principal component	1.20 (1.01-1.42)	.04
Perceived cancer screening self-efficacy score (range, 4-20)	1.07 (1.002-1.14)	.047
National percentile of block group ADI score	0.99 (0.98-0.998)	.03

Abbreviations: ADI, area deprivation index; DVD, digital video disc; FOBT, fecal occult blood test; OR, odds ratio; PN, patient navigation.

^a^ Reconfirmation of medical record location was also adjusted for in this model (confirmed vs not confirmed at 12 months: OR, 4.55; 95% CI, 2.56-8.33; *P* < .001).

^b^ Effect of DVD/PN vs DVD: OR, 2.98; 95% CI, 2.09-4.18; *P* < .001.

Participants who perceived their finances as inadequate to pay their bills were half as likely (OR, 0.45; 95% CI, 0.24-0.81; *P* = .01) to be up to date for any needed cancer screenings compared with those who reported having enough money to pay their bills. Participants who were working full time compared with those not working were more likely (OR, 1.58; 95% CI, 1.07-2.36; *P* = .02) to be up to date at 12 months for any cancer screening (Table 4). Participants who intended at baseline to obtain needed screenings in the next 6 months (OR, 1.85; 95% CI, 1.33-2.59; *P* < .001), those who had higher knowledge (OR, 1.20; 95% CI, 1.01-1.42; *P* = .04) and self-efficacy (OR, 1.07; 95% CI, 1.002-1.14; *P* = .047) scores, and those who had lower ADI scores (OR, 0.99; 95% CI, 0.98-0.998; *P* = .03) had greater odds of being up to date for screening for any cancer. Higher perceived barrier scores to screening were associated with higher odds of completing screening (OR, 1.23; 95% CI, 1.03-1.47; *P* = .02), although there was no interaction with the intervention. Age of 65 years or older was not associated with being up to date for any screening outcome (OR, 0.91; 95% CI, 0.55-1.49; *P* = .70).

### Sensitivity and Cost Analyses

The intervention effectiveness (eg, ORs and *P* values) did not change meaningfully in our sensitivity analysis when all participants aged 66 years or older were considered as being up to date at baseline with cervical cancer screening for analyses of 12-month all or any cancer screening test up-to-date outcomes (eTables 2 and 3 in Supplement 2). We conducted a cost analysis to determine the additional costs associated with each additional unit of being up to date for all screening tests gained from the (1) DVD intervention and (2) DVD/PN intervention compared with the usual care approach, which had no incremental costs (eAppendix in Supplement 2). Excluding research costs, we found a total cost of $326 012 for the DVD intervention and an additional $344 829 to add patient navigation to the DVD intervention. Normalizing on the main outcome of being up to date with all needed screening tests, the cost-effectiveness amounted to $14 462 per up-to-date participant in the DVD group and $10 638 per up-to-date participant in the DVD/PN group.

## Discussion

The goal of this study was to compare the effectiveness of 2 interventions with usual care to increase the proportion of rural women up to date with screening for 3 cancers (breast, cervical, and colorectal). We considered the following 12-month outcomes: being up to date with all screening tests and being up to date with any needed screening tests (ie, for 1, 2, or 3 cancers depending on baseline status). Although other studies have been successful at simultaneously increasing both colorectal and breast cancer screening or cervical and breast cancer screening,^15,17,23^ to our knowledge, interventions to increase the uptake of screening for 3 cancers simultaneously have not been tested. Our findings demonstrate that interventions delivered remotely to rural women can simultaneously improve screening rates for breast, cervical, and colorectal cancer. Following the mailed DVD, participants in the DVD/PN group received patient navigation to reduce their individual barriers to needed cancer screenings. While participants receiving only the DVD intervention were almost twice as likely to be up to date with all cancer screenings, the addition of a patient navigator was almost 6 times more effective than usual care, supporting the importance of patient navigation.

Participants had greater odds of becoming up to date with all screenings if only 1 screening was needed or if breast cancer was 1 of the 2 screenings needed. Within our study, when only 1 screening is needed, it is easier for women to become up to date with all screenings. If a participant needed screening for more than 1 site, the intervention was more intense because it focused on obtaining any cancer screening test that was not up to date. Although the need for multiple screening tests could have increased the time needed for intervention activities, consolidating efforts was still more time efficient than addressing 1 needed cancer screening test at a time and probably at different times. Additionally, our baseline breast cancer screening up-to-date rates were higher than baseline rates for cervical and colorectal cancer screening, suggesting that this population of women may find it easier to become up to date with mammography vs cervical or colorectal cancer screening. Consistent with this finding, previous studies have found higher rates of breast cancer screening compared with colorectal or cervical cancer screening.^50^ It is difficult to determine why the study found that women who were due for mammography compared with cervical or colorectal cancer screening were more likely to become up to date for all tests. The fact that mammography screening is discussed more in the media might make it more socially acceptable than the other cancer screening tests.^51^

Sociodemographic characteristics associated with becoming up to date with screening tests were similar to reports from previous studies.^52,53,54,55^ Full-time employment is often linked to health insurance, and both have been consistently shown to be associated with being up to date with all 3 cancer screenings.^52,53,54^ Compared with younger participants, those 65 years or older were only half as likely to be up to date with screenings for all cancers at 12 months. However, age was not associated with being up to date for any screening outcome. Regarding the significant inverse relationship between age and all screening outcomes, the literature has indicated that adherence to needed screening tests increases with age.^55^ Being up to date with all needed tests could be more problematic for older women, as there might be more barriers (practical and personal) in this age group to completing multiple screening tests within a 12-month period.

Among our theoretical measures, intention to screen, knowledge, and barriers reported at baseline were related to becoming up to date at 12 months after randomization. A participant who self-reported intention (contemplation) to screen in the next 6 months had almost 2 times the odds of becoming up to date with all needed cancer screenings, consistent with prior research based on the transtheoretical model of behavior change.^56,57^ Consistent with other studies, greater knowledge was related to becoming up to date with screening tests.^58,59^ Unlike in other studies,^59,60^ higher scores of perceived barriers at baseline were associated with being up to date for any cancer screening at 12 months. Patient navigator calls focused on reducing barriers that might keep women from engaging in needed screenings; thus, participants with more barriers may have experienced increased interaction with the patient navigator.

The USPSTF guidelines support that cervical cancer screening not be done after 65 years of age if results in the past 3 years were negative.^25,26,27^ Sensitivity analyses (eTable 2 in Supplement 2) revealed that when participants aged 66 years or older were all considered up to date at baseline based on current cervical cancer screening guidelines,^11^ the results showed similar intervention effects as observed in the primary analyses.

The DVD/PN intervention was more cost-effective in bringing participants up to date with all needed tests due to the greater effect size. Compared with treating cancer, the costs of each intervention to bring women up to date with screening were relatively modest, as on average, cancer treatment costs $150 000 per patient in the US,^61^ and costs of the intervention would be lower per person at a larger scale. Thus, the additional costs required for the addition of PN to improve screening may result in cost savings by avoiding cancer deaths or treatment at more advanced stages.

### Strengths and Limitations

This study had some strengths and limitations. Our sample was highly educated and predominately White, making translation to a less educated and more diverse population difficult.^62^ However, our study counties have few racial and ethnic minority residents. Although 84% of our population had some college or higher, the DVD technology was completely narrated, rendering it accessible regardless of educational attainment. We found that all participants had the requisite technology necessary to use the interactive DVD, although this technology is rapidly becoming obsolete, creating the necessity to translate the intervention to an online tool that can be accessed via a computer, tablet, or smartphone. This intervention was delivered to rural women who, at the time of the study, had limited internet access; therefore, remote delivery was best suited to DVD technology.^63^ This study supports the one-stop-shop approach as advocated by other researchers who also found that a screening intervention could simultaneously improve the uptake of more than 1 cancer screening test. The potential for increasing multiple screening behaviors at one time is especially relevant for rural communities where health care may be hampered by remote living conditions that limit access to preventive services.^15,16,17,18,19,23^

## Conclusion

In this randomized clinical trial, a single intervention was used to support being up to date for any or all USPSTF guideline–supported screenings for women (breast, cervical, and colorectal cancer) aged 50 to 74 years. The effectiveness of these interventions that targeted all or any needed cancer screenings simultaneously offered an approach that can be delivered remotely to rural women and has paved the way to approach preventive health care holistically, fostering cancer prevention and early detection when a cure is realistic and ultimately decreasing cancer health disparities.

## Notes

Supplement 1.
Trial Protocol

Supplement 2. **eTable 1.** Bivariate Analysis of Baseline Covariates and Screening Outcomes **eTable 2.** Logistic Regression for All Cancer Screenings at 12 Months Postbaseline (n = 852) **eTable 3.** Logistic Regression for Any Cancer Screening at 12 Months Postbaseline (n = 847) **eAppendix.** Intervention Cost Methods

Supplement 3.
Data Sharing Statement
