# Supplementary material for: Comparative Effectiveness of 2 Interventions to Increase Breast, Cervical, and Colorectal Cancer Screening Among Women in the Rural US: A Randomized Clinical Trial
Source: JAMA Netw Open. 2023 Apr 28;6(4):e2311004. doi: 10.1001/jamanetworkopen.2023.11004 (PMC10148202; doi:10.1001/jamanetworkopen.2023.11004)
Supplement: Supplement 1. — Trial Protocol [file jamanetwopen-e2311004-s001.pdf]

SPECIFIC AIMS

In 2014, approximately 310,030 women in the United States (U.S.) will be diagnosed with breast cancer (BC), cervical cancer (CC), or colorectal cancer (CRC), and 68,060 women will die from one of these cancers.[2] Combined, these three cancers account for 39% of the cancer burden and 25% of the cancer mortality among U.S. women. Although screening tests for these cancers are widely available, many women do not adhere to screening guidelines.[3-5] The counties in northwest Ohio (OH) and northeast Indiana (IN), share a similar socioeconomically underserved population of rural women who have elevated BC, CC, and CRC mortality rates and lower screening rates (Tables 1 & 2).[6-9] This application focuses on this <u>underserved rural population</u> of women (map in Resources and Environment; Figure 1).

Table 1. Cancer Mortality Rates: Rural Indiana, Rural Ohio[1], and the United States (U.S.), Females.[2]

Cancer Site	Rural Indiana	Rural Ohio	U.S.
**Breast**	23.9	23.4	22.7
**Cervical**	2.4	2.8	2.1
**Colorectal**	17.4	18.9	16.4

[1]Age-adjusted mortality rates (2007-2011)

[2]Age-adjusted mortality rates (2006-2010)

Prior theory-based interventions to improve cancer screening among rural women are limited, and <u>no known intervention has addressed improving screening rates for these three main female cancers simultaneously</u>, i.e. testing multiple health behavior change for these three screening tests.[10-12] This area of research is emerging as a new model to change the way interventions can be packaged, and is consistent with an emerging paradigm of preventive health.[12,13]

Table 2. Estimated Screening Test Prevalence (%) for Women by County Classification, State (IN and OH) and U.S

	Rural IN	Rural OH#	IN	OH	U.S.
Mammogram in last 2 years*	67	74	70	77	77
Pap Test in last 3 years**	69	73	73	79	78
Colonoscopy/FS Ever **	56	63	64	67	69

**Women 50 and older; **Women 18 and older; # Excludes Appalachia; IN=Indiana; OH=Ohio* Source: Behavioral Risk Factor Surveillance System Survey, 2012, and Bureau of Health Ohio, 2014; Behavioral Risk Factor Surveillance System Survey, 2012, Indiana State Health Department, 2014.

This proposal will test the <u>comparative effectiveness</u> of a tailored interactive computer program delivered via DVD (TIDVD) vs. a TIDVD plus telephone-based patient navigation (PN) intervention (TPN) vs. Usual Care (UC) to increase guideline-based screening rates for BC, CC, and CRC among women age 50 to 74 living in socioeconomically depressed rural counties of northwest OH and northeast IN. Building upon our prior work, we include a multi-institutional health disparities team that has experience in interventional cancer screening research, underserved and rural populations, tailoring interventions, and PN research.

The specific aims are to:

Aim 1: Compare the effectiveness of a tailored and interactive DVD (TIDVD) vs. a TIDVD plus telephone-based PN intervention (TPN) vs. Usual Care (UC) to increase guideline-based cancer screening rates for BC, CC, and CRC at 12 months via Medical Record Review (MRR) among 1058 women age 50 to 74 living in rural northwest OH and northeast IN.

<u>**Primary Hypothesis**</u>: Women in the TIDVD+TPN group will have higher rates of within guideline adherence to <u>all screening tests</u> via MRR at 12 months compared to those who receive the TIDVD alone or UC.

<u>**Secondary Hypothesis:**</u> Women in the TIDVD+TPN group will have higher rates of within guideline adherence to <u>any</u> needed (CC <u>or</u> BC <u>or</u> CRC) screening test compared to those who receive the TIDVD alone or UC, to allow for assessment of the intervention effectiveness by type of screening test.

Aim 2: Compare the <u>cost-effectiveness</u> of the TIDVD compared to the TIDVD+TPN intervention for adherence to <u>each</u> screening test outcome or <u>combination</u> of screening tests, as defined in the primary and secondary hypotheses.

<u>**Hypothesis:**</u> The TIDVD + TPN intervention will be more cost-effective than the TIDVD intervention or UC for adherence to each or combination of screening tests.

Exploratory Aim: Identify associations between theoretical variables (community, social, and individual) and screening test adherence, including interactions with the interventions.

IMPACT: If found to be cost effective, either or both interventions have the potential to be immediately disseminated to the underserved population of rural women in the U.S., thus significantly improving BC, CC, and CRC screening rates and ultimately reducing cancer disparities in screening rates and mortality. <u>This study is among the first to target a rural population with demonstrated differences in cancer mortality and addresses three cancer screening tests simultaneously, using an innovative combination of interventions.</u>

RESEARCH STRATEGY

1. Significance

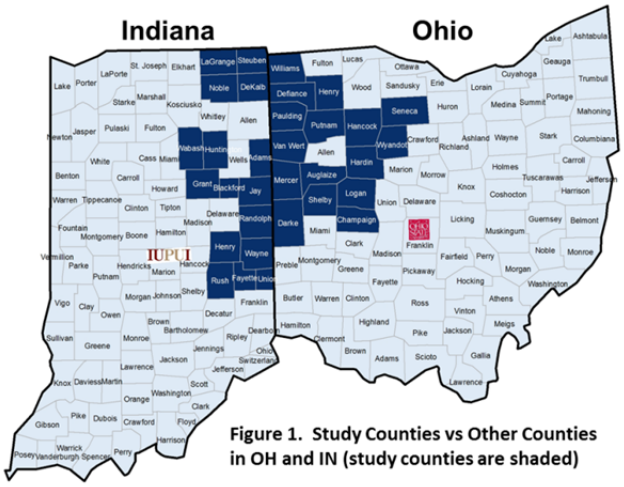

Figure 1. Study Counties vs Other Counties in OH and IN (study counties are shaded)

The cancer burden among rural and underserved populations is significant. Residents living in rural areas in the U.S. experience higher cancer incidence and mortality rates, as well as lower screening rates.[8,9,14-16] The breast cancer mortality rate in our Ohio and Indiana counties are 9.7% and 5.8% greater, respectively, than that for the U.S., while the cervical cancer mortality rate is 12.5% greater, and the CRC mortality rates are 10.4% and 6.1% greater, respectively, than that for the U.S. This results in 106 excess deaths in these counties per year. Reasons for cancer disparities among rural populations are due to many social determinants of health including lower socioeconomic status (SES), lower educational levels, lifestyle factors, genetics, limited access to healthcare, lack of health insurance, or a combination of these factors.[14-17] Screening adherence rates for BC, CC, and CRC cancers, for which there are validated screening tests available, are lower for those with less education, a proxy for SES.[3,18] The rural counties targeted for this application (Figure 1), include mainly White, poorer, and less educated populations with limited access to health care. Thus, it is essential to intervene in these rural counties to reduce cancer disparities.

Interventions to improve screening rates need to be extended to rural populations. While our investigators have led some of the major efforts in developing and testing interventions to improve cancer screening among White, African American, and rural Appalachian populations, we have not extended this work to the rural, non-Appalachian population in our states.[7,19-28] Many rural women do not complete screening at recommended intervals.[16] We just completed a study among rural women in Ohio and medical record validated completion of screening within recommended guidelines was: 32% for mammography, 36% for Pap test, and 30% for a CRC test.[73] Only 8.6% had completed all three tests within guidelines.[ref] This suggests the need for cost effective interventions to provide rural women with the latest information about screening recommendations and needed tests.[29,30]

The significance of this study is enhanced by the potential to develop a stronger more efficacious intervention through the combination of two previously tested interventions (tailored interactive programs and patient navigators). This research builds on the prior research of both investigators (Paskett and Champion) by testing the hypothesis that the addition of a Patient Navigator (PN) to the tailored program will be more effective than a tailored approach alone or UC. The investigative team assembled for this project has led some of the most comprehensive investigations of the efficacy of tailored programs using a DVD and PN interventions completed to date.[20,25-28,31-33] IU investigators have used TIDVD to improve BC screening in insured and underserved populations, while the OSU team has demonstrated the effectiveness of both PN[31] and other types of lay “natural helper” change agents (e.g. Lay Health Advisors, LHA’s) to improve BC, CC and CRC screening in rural and underserved/minority populations (Section 3.3). Our pilot data suggest that combining a LHA intervention with a tailored computer program can significantly increase BC screening.[32]

Finally, this proposal is greatly enhanced by the comparison of the cost-effectiveness of interventions that vary in complexity, time, and cost. We will determine if adding a telephone-based PN (TPN) will be worth the additional costs by comparing the TIDVD intervention with and without TPN vs. UC. If either or both interventions are shown to be cost-effective compared to UC to improve screening rates, they can be quickly/easily disseminated to rural areas where access to health care is suboptimal, and cancer mortality rates for CC, BC, and CRC are higher than non-rural areas.

2. Innovation

The current proposal supports four innovative strategies to further the knowledge and translation of cancer screening interventions. ***First,*** though numerous studies have tested interventions for cancer screening, we will be the first to develop an intervention that simultaneously targets the **three** most important cancer screening tests -cervical, breast and colorectal- for understudied women in rural areas. Interventions that promote adherence to multiple cancer screening tests (e.g. >2 tests) simultaneously have tremendous potential to reduce the disproportionately high BC, CC, and CRC mortality rates for rural, predominantly white, underserved women.

***Second*, our study is the first to test a combined tailored and TPN intervention to increase cancer screening behaviors in rural populations.** To our knowledge, a tailored intervention plus PN has not been tested to improve multiple cancer screening tests. Thus, testing the combination of intervention represents an innovative comparative effectiveness study; a one-stop approach to improving adherence to the three major cancer screening tests.

***Third*, our theoretical framework includes individual, social, and community level variables that may impact intervention efficacy and cost-effectiveness.**[34] It is essential when considering translation that we identify moderators and mediators of intervention effectiveness at multiple levels to increase the potential efficacy and translation of interventions. Building on the investigators' previous research (tailored DVD and PN), we will tailor interventions for BC, CC, and CRC based on a woman's individual need for screening, taking into account individual, social and community level factors that impact these behaviors.

***Fourth*, few interventions to increase screening have targeted rural populations which suffer from health disparities especially cancer mortality.** We will use the underserved rural populations in our respective states to determine the effectiveness of this intervention approach in a rural population which has not been a focus of many previous studies. Knowledge from this proposal has the potential to strengthen current screening interventions and test their applicability and translation to underserved rural women.

3. Approach

3.1. Theoretical Framework of Study

In recent years, our understanding of disease pathways (causes and progression) has broadened considerably to include acknowledgement of the important roles played by psychosocial, economic, social, environmental, and institutional factors. An integrated conceptual model is the foundation for understanding and influencing women's cancer screening behaviors. We will use individual, interpersonal, social, and community level constructs to better understand the disparities in our population, and to help understand how these factors impact the effectiveness of the interventions being tested.

Figure 2. Conceptual Model

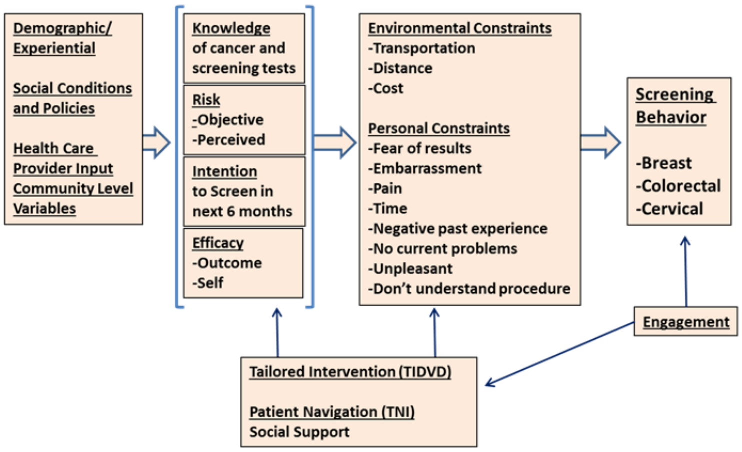

3.2. Conceptual Model for the Intervention

A multiple health behavior change intervention is especially appealing when addressing behaviors that are conceptually similar-such as cancer screening behaviors. Cancer screening behaviors have many commonalities and have been found to be correlated with common variables predicting screening for all 3 tests.[13] For example, the theoretical principles used to make behavior change for each cancer screening test include self-efficacy, and the use of change processes that move an individual through stages of change.[35-37] The interventions proposed will focus on the needed cancer screening tests concurrently and teach principles of behavioral change (e.g. self-efficacy). Several studies have found that interventions targeting multiple behavior change (but none have focused on 3 screening test behaviors) have been effective in simultaneously changing up to five behaviors. In 2005, a randomized controlled trial was effective in helping CRC patients change multiple behaviors.[74] More recently, researchers found that an intervention targeting multiple behaviors was successful in decreasing sedentary behavior in CRC survivors.[75] Johnson, coined the word coaction to reflect the synergistic effect one behavior can have on another.[76] He concluded that regardless of study design or other variability, coaction was consistently found in multiple behavior studies.

The conceptual model (Figure 2) uses constructs from the Theory of Reasoned Action, Theory of Planned Behavior, as well as other well-established behavioral theories.[38-41] This model will build on previous work by identifying an individual's beliefs about variables known to predict behavior change.[38,42,43] These variables will have intervention messages tailored to each women's input. Our goal is to provide a unified intervention that supports adherence to all 3 cancer screening tests, depending on individual screening needs. Responses are tailored to specific tests needed by the individual. Ability to carry out tests will vary based on which test is being discussed and the program will tailor messages appropriate to the test(s) needed.[42-44] Variables that may affect beliefs directly or indirectly such as demographics, health care provider input, and social conditions and policies identified in Figure 2. This model will be used to identify intervention effects and to test model development. Intervention "engagement" has been identified by researchers and theorists as a critical component of intervention effectiveness and will be measured and included in the analyses.[45]

3.3. Previous Studies

The proposed study represents the logical next step in our active programs of research to test innovative approaches to increase screening among underserved populations. This highly qualified team of investigators from OSU and IU has a strong history of collaboration and extensive experience conducting behavioral interventions (using LHA's, PN's, and computer-tailored interventions), and health services research.

3.3.1. OSU Team. Drs. Paskett and Katz, bring behavioral science expertise to the partnership. PN: Dr. Paskett was the lead investigator of one of ten sites that comprised the National Patient Navigation Research Program (ACS Grant SIRSG-05-253-01; Co-I's Katz, Seiber, Young, Tayal) to test the effectiveness and cost of PN for patients with abnormal screening results for BC, CC and CRC. The study showed that PN significantly improved resolution of abnormalities.[31,33] Similar data collection methods, instruments, recruitment procedures, and PN training protocols will be utilized for this study. The OSU team has conducted research testing behavioral interventions among minority and underserved populations to improve cancer screening rates, using LHA interventions. Dr. Paskett completed studies among low-income urban populations to improve BC and CC screening,[22] among a rural tri-racial population to improve BC screening,[21] among minority women in rural/urban NC and SC with Dr. Katz to improve CRC screening,[46] and among Ohio Appalachian women in need of CC screening.[20] Dr. Katz conducted a study using patient activation to improve CRC screening among a minority and low SES population (K07 CA107079; Mentor: E. Paskett).[23] Drs. Paskett/Katz are testing clinic-based interventions with PN (R01CA116487; Co-I: Young) and community-based interventions among rural Appalachian communities (R24MD002785) to improve CRC screening.

3.3.2. IU TEAM. Drs. Champion, Rawl, and Monahan have over 20 years of experience developing and testing interventions to increase cancer screening, with specific expertise in developing computer-tailored messages and using interactive media to test interventions.[24,27,28,47,48] The IU team has tested numerous technologies to deliver computer-tailored interventions via print, telephone, tablet computers, interactive touch screen computers, and mailed DVDs. Our most recent studies have used computer based algorithms to deliver tailored interventions by testing a tailored DVD program against usual care and tailored telephone intervention. Drs. Rawl and Champion completed a study testing a tablet-based computer-tailored intervention to promote CRC screening among African American patients in primary care settings (R01-CA115983, PI: Rawl). At 6 months, those who received the computer tailored intervention were significantly more likely to have completed CRC screening than patients who received a standard CRC screening brochure.[28] Most recently the same team tested a tailored program breast cancer screening intervention delivered through a mailed DVD. Women who received the DVD and had a household income below $75,000, received significantly more mammograms compared to usual care (adjusted odds ratio = 1.51,p=.017).[49] We also found that 92% of women (n=926) who received a mailed DVD reported using the program and rated it high on a usability scale (1 R01 NR008434).[48]

3.3.3. OSU and IU Collaborations. Dr. Paskett and Champion collaborated with Dr. Kathleen Russell on a KO1 (K01 CA111826) to test the comparative effectiveness of an interactive computer program to improve BC screening (developed by Dr. Champion[50]) vs. the program plus a LHA intervention (developed by Dr. Paskett[21]). This study demonstrated the effectiveness of the combined interventions on improving BC screening in low-income African-American women from community settings.[32] Drs. Paskett and Katz have collaborated with Drs. Champion, Rawl, and Monahan on a PO1 application (PI: Champion), and a RO1 application (PI: Rawl) specifically to deliver PN interventions for CRC screening and follow-up, among low-income African-Americans.

3.3.4. Pilot Feasibility Study. In preparation for the submission of this proposal, the investigators conducted a small pilot study in conjunction with Dr. Paskett's ongoing study in rural Ohio (R24MD002785). In the parent study (N=1,091), as confirmed by MRR, 90% of the women needed at least one of the 3 screening tests and the distribution of tests needed is shown in Table 3. For the pilot study, randomly selected women aged 51-74 who were drawn from commercial lists (Salesgenie; commercial plus USPS lists) and lived in one of 12 Appalachian study counties in OH, were sent a letter, consent form, survey and URL where they could view an interactive tailored web based program. The program promoted cancer screening but used a Web based program instead of the DVD media described by Champion, 2014. Women were instructed to view the program, and return the consent form and completed survey. Five days later, a PN called each woman to assess barriers to receiving BC, CC and CRC screening tests, as appropriate, collect information on intent to receive needed

Table 3. Possible Strata By Screening Status At Baseline

Adherent at baseline to:	Group A	Group B	Group C	Group D	Group E	Group F	Group G
CC screening	No	Yes	Yes	No	Yes	No	No
BC screening	No	No	No	Yes	Yes	No	Yes
CRC screening	No	No	Yes	Yes	No	Yes	No
Distribution	45%	12%	6%	8%	10%	11%	7%

screening tests, and experiences with the interactive program. Of the 40 women selected to receive a survey, 5 were ineligible to participate (12.5%), and among those eligible, 66% completed the survey. All women who viewed the Web-based reported liking the intervention and 80% indicated that they would get at least one needed test. Of those women who had not viewed the program (7), only 57% indicted they would get at least one needed test. While only 65% of women reported viewing the program, this was attributed to a limited amount of time before being called by the PN and the fact that some women may not have access to high speed internet. As described above, a similar tailored program was delivered by a mailed DVD, resulted in 92% of women viewing the intervention.[49] The difference in utilization between the web-based program and DVD, supported our decision to deliver the proposed intervention by mailed DVD. The pilot confirmed that a bundled intervention is feasible, will be well-received in a rural population of older women using a tailored methodology, and has the potential to increase screening rates.

Table 4. United States Preventive Services Task Force Screening Guidelines

Site	Guideline
BC	Women ages 50-74: mammogram every 2 yrs.
CC	Women ages 50-65: Pap test every 3 yrs. or Pap test and HPV co-testing every 5 yrs. Discontinue after age 65 for women with 3 consecutive negative cytology results or 2 consecutive negative HPV results in last 10 yrs.
CRC	Women ages 50-75: stool test (FOBT/FIT) annually or colonoscopy every 10 yrs. For those at increased risk; colonoscopy is recommended.

3.4. Setting. Rural Ohio and Indiana

Both OH and IN have sizable populations of rural residents (23% OH and 29% IN), and the mortality rates for the 3 targeted cancers are higher among these rural residents.[51] Our study will target women aged 50 to 74 who reside in 32 rural counties in northwest OH and northeast IN (map in Resources and Environment; Figure 1). In these counties (2010), there were 173,812 women in the appropriate age category, 96% who are White (non-Hispanic). We will sample 1058 women 50-74 years in the designated counties, stratified by Rural Urban Commuting Area (RUCA) code (4-6 vs. 7-10; Section 3.9.1.).

3.5. Participants

3.5.1. Sample Eligibility. To be eligible, women must: 1) be aged 50-74 years; 2) be non-adherent to one or more recommended screenings for BC,CC, or CRC by MRR; 3) reside in one of 32 rural counties in IN or OH; and 4) provide consent. U.S. Preventive Services Task Force cancer screening guidelines will be used to determine eligibility for all screening tests (Table 4).

Over 96% of these women are non-Hispanic. Spanish-speaking women will not be excluded, as we will use our Spanish-speaking staff to administer consent and the surveys and for PN. We will assist with translation of the DVD and provide comparable mailed materials in Spanish. If more than one female is eligible in a household, we will include the person with the closest birthday from date of the call. Women will be excluded if they have: 1) a personal or family history of any hereditary/genetic cancer syndrome such as BRCA1 and BRCA2 polymorphisms, hereditary nonpolyposis colon cancer, or familial adenomatous polyposis; or 2) inflammatory bowel disease (Crohn's disease), colon polyps, or a history of cancer except non-melanoma skin cancer. Based on data from our pilot study (Section 3.3.4) that included rural women in this age group recruited in a similar manner to the proposed sampling plan (R24MD002785), we estimate that approximately 10% of those approached will be within guidelines for all three tests, by MRR (see Table 3). Using those eligibility criteria, we estimate that 55% of those contacted will be eligible, and 70% will participate, based on our prior studies among rural women.[52]

3.5.2. Sample Size. Estimates of effect size (Table 5) for the intervention at 12 months is based on the PIs' experience in projects promoting screening among underserved populations. We estimate 10% attrition at 12 months, when our primary outcome will be assessed. Attrition of 10% is conservative because we will have medical record data on most of the persons who drop out which will reduce the effect of drop out on the primary outcomes. We based power calculations on 2-sided tests when comparing the two intervention arms (TIDVD vs. TIDVD+TPN) because we could not be certain which group would perform better. Because of the overwhelming evidence in the literature[20,49] that the UC arm does not have better screening rates than tailored DVD or PN interventions, we based the power calculations on 1-sided tests for comparisons to the control group (TIDVD vs. UC; TIDVD+TPN vs. UC). The planned statistical analyses are consistent with these power calculations. Specifically, we will use two sided tests for TIDVD vs. TIDVD+TPN, and the 1-sided tests for comparing interventions to the UC arm, using the 1-sided p-value from the Z statistic in contingency table analysis and from the likelihood ratio test in logistic regression analysis. To achieve 80% power for the contingency table analyses, and the likelihood ratio tests of logistic regression for the primary outcome of adherence to all needed screening tests at 12 months by MRR, a sample size of 356 per each intervention

group and 180 in the UC group will be needed, which will require 396 per intervention group and 200 in the UC group at baseline to account for attrition. For 80% power for the secondary outcome of adherence to any needed (BC <u>or</u> CC <u>or</u> CRC) test, regardless of the number of screening tests needed, a sample size of 376 per each intervention group and 200 in the UC group will be required at 12 months. We will require 418 per intervention group and 222 in the UC group at baseline to account for attrition and to achieve at least 80% power for both outcomes. The total sample size is 1058 participants enrolled with <u>418 randomized to each intervention arm and 222 randomized to the UC arm.</u>

Table 5. Sample Size*

Effect sizes for screening at 12 months	Control (UC) 12 Months	TIDVD 12 months	TIDVD+ TPN 12 months	N needed per TIDVD and TIDVD+TPN at baseline for 80% power at 12 months	N needed in control arm (UC) at baseline for 80% power at 12 months	Projected N per TIDVD and TIDVD+TPN intervention arms at 12 months	Projected N in control arm (UC) at 12 months	Power TIDVD vs. TIDVD+ TPN	Power TIDVD vs. control (UC)	Power TIDVD+ TPN vs. Control (UC)
**Cervical**	35%	50%	70%	NA	NA	NA	NA	NA	NA	NA
**Breast**	35%	45%	65%	NA	NA	NA	NA	NA	NA	NA
**Colon**	30%	40%	55%	NA	NA	NA	NA	NA	NA	NA
**All needed screenings (primary outcome)**	10%	20%	30%	396	200	356	180	82%	83%	99%
**Any (at least 1) needed screening (secondary outcome)**	25%	35%	45%	418	222	376	200	80%	80%	99%

*Estimates of effect sizes for the secondary outcome (at least 1 needed test adhered to by 12 months) was based on the most conservative single test (i.e., colon cancer screening). Effect sizes for the primary outcome were based on an estimated reduction of 5% in the number of participants who would adhere to all needed tests compared to only one of the needed tests. Power columns are based on the most stringent required sample size, i.e., when N=418 per each intervention arm and N=222 controls at baseline and projected 376 per each intervention arm and 200 controls at 12 months.

3.6. Overview and Design

In Year 1, we will convene the Community Advisory Board (CAB), refine and pilot the interventions and UC materials with 20 rural women, modify the interventions, refine our sampling process, train the PNs, collect information on local resources for screening and follow-up, and finalize all study materials, including study questionnaires. The full study, designed as a randomized controlled trial (Figure 3), will begin in Year 2.

Figure 3. Study Schema

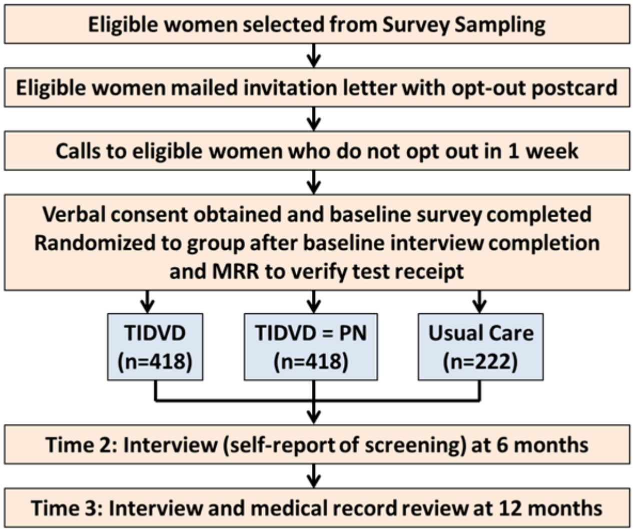

3.7. Community Advisory Board (CAB)

We will convene a new CAB with members from the investigators' prior CABs and add representative community leaders and residents from the study counties (see letters of support). Community leaders, e.g. County Commissioners, leaders of local churches, members of the local women's groups, will also be contacted and invited to participate, as well as consumers and women from the respective counties who are cancer survivors. The local American Cancer Society (ACS) offices, extension offices, and the OSU and IU investigators will be instrumental in identifying additional members of this advisory board. Drs. Paskett and Katz have experience working with a CAB for the past decade that includes members from five states in the Appalachia Community Cancer Network (ACCN). These CAB members have had equal input into study-related issues without any difficulties or challenges across 5 states. Thus, we feel that a 2-state CAB, as we propose, will be feasible and productive.

To address the geographic distance of the study area, we will rotate our meeting sites and allow members who cannot travel to participate by phone. The CAB will meet two times during each year of the project. The CAB will assist in not only making our interventions attractive and relevant to the communities we will work in, but in sustainability and dissemination of efficacious interventions. The duties of the CAB will be to strengthen our relationship with the rural communities through door-opening and building on strengths and resources within the communities. The CAB will review interventions, sampling strategies, and recruitment to provide feedback to the study investigators and staff.

3.8. Interventions

Table 6. Sample TIDVD Messages Tailored to Screening Adherence at Baseline

<u>Needs only CRC screening</u>	<u>Needs BC and CRC screening</u>	<u>Needs BC, CRC, and CC</u>
You already understand the benefit of mammography and Pap tests but might not know that CRC screening benefits you in the same way. Just like BC or CC, if CRC isn't found early, it can spread to other parts of your body making it hard to treat and may cost you your life. The good news is that all cancers can be found early – before they spread and when almost all women are completely cured.	You already understand the benefit of having Pap tests but might not know that BC and CRC screening benefits you in the same way. If BC or CRC isn't found early, it can spread to other parts of your body making it hard to treat and may cost you your life. The good news is that these cancers can be found early – before they spread and when almost all women are completely cured.	Many women don't realize that BC, CRC and CC all start as a single cell that grows and divides and then and then turns into cancer. If these cancers aren't found early, they can spread to other parts of your body and may cost you your life. The good news is that these cancers can be found early – before they spread and when almost all women are completely cured.

This study will test two interventions (TIDVD and PN) in comparison to UC. The following sections describe the two interventions separately as well as the intervention pre-testing. Both interventions (TIDVD and TPN) will address any combination of screening tests needed by an individual woman. As seen in Table 4, women will be categorized into one of seven groups (Group A through Group G), based on baseline adherence status for each of the three screening tests. The ability of either the intervention arm to simultaneously address any combination of the three screening tests (BC, CC, or CRC) represents a holistic approach to preventive care that may be more efficacious and cost effective. For example, in women who need both mammography and CRC screening (Group B), both the TIDVD and TPN interventions will provide messaging about the common benefits of screening for only CRC and BC, but build on the success of CC screening (Table 6).

If a woman indicates <u>abnormal</u> <u>signs or symptoms</u> at any time,

she will be referred to her health care provider or a health care clinic. We will identify any <u>abnormal screening tests</u> that occur in the follow-up surveys and a PN will contact women (in any study arm) to assure they are able to follow-up with further testing. Since this will be done after the intervention is concluded and outcome data (screening adherence) are collected, this action will not interfere with the study results. The two interventions, TIDVD and TPN, are described below.

3.8.1. Tailored DVD. The TIDVD will be refined from an existing TIDVD developed by Drs. Champion and Rawl.[49] Refinement of the current TIDVD will include addition of a cervical screening component and the addition of barriers unique to rural women that are identified during focus groups. We will create a TIDVD that can be mailed to a participant and played on a regular DVD player. The participant interacts in real time with the DVD by answering questions posed by the DVD program and using arrow keys on the remote to highlight and enter their response (Figure 4). All possible tailored messages and videos are included in the TIDVD program and algorithms program messages and videos tailored the individual's answers to questions. The TIDVD format was selected for three reasons. *First*, the majority of women in rural areas have a DVD player. In preparation for this grant submission, we surveyed 72 rural women aged 50-74 about their access to a DVD player and (92%) stated they had a DVD player or access to a personal computer with a DVD player. <u>These data indicate our TIDVD-based intervention is feasible in this population</u>. *Secondly*, using the remote, women can answer questions allowing the delivery of tailored messages in real time (Figure 4). A DVD program can deliver health messages with visual aids such as graphs, video clips, and storytelling. Additionally, interactive programs enhance personal engagement in the intervention, thereby increasing the effect of the intervention, as described in the theoretical context.[49] Once data are obtained from the individual, the program delivers a message developed specifically for the data provided in real time. For example, a message about the barrier of embarrassment with CRC screening would be delivered if the participant indicated she was embarrassed about getting CRC screening. Research has demonstrated that the largest effect sizes for tailored interventions are obtained when tailoring on 4 or more concepts or demographic variables, thus we are tailoring on multiple barriers, as well as age and rural residence.[39,40,56] *Third,* DVDs can be duplicated quickly with minimal expense and mailed to participants. The tailored program begins with a generic introduction that explains the use of the DVD program and asks a few basic questions to determine adherence to each of the 3 screening tests, as well as age (in decade), race, and family history. Responses to these questions are stored in memory and integrated into responses (obtained throughout the program) to queries about belief constructs (perceived risk, benefits, and self-efficacy). Throughout the TIDVD program, women receive programmed tailored messaging bundled to their screening status on CC, BR and CRC (Table 7). Risk messages are tailored to both perceived and objective risks. For barriers, a list of potential barriers will be presented and women will indicate up to three barriers for the relevant test. Barriers will be vetted by focus groups in the targeted population. The DVD program ends with the narrator encouraging viewers to make an appointment for any of the screenings needed. On average, our previous interactive DVD program required 10 minutes to complete. The proposed study will modify the original program to include a cervical screening segment (R01 NR008434, PI Champion). All visual screens are narrated with

Figure 4. Remote

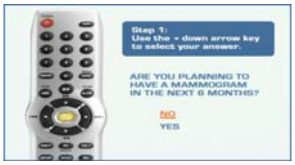

the critical information appearing as written text, allowing women with low literacy to use the program (Appendix B: example using CRC screening).

An experienced professional multimedia development team from the IU School of Informatics will edit and revise our current BC and CRC intervention and add the CC screening intervention piloted for this application. These refined prototype interfaces will appear as complete designs. Program coding will be done using DVD Studio Pro. The prototype will be tested in focus groups of rural women (Section 3.8.3).

3.8.2.a. Tailored DVD + TPN. The second intervention group will receive a TIDVD intervention followed by a telephone call from a PN. PN interventions have been utilized in all types of populations, including low-income and minority populations. Many types of people are used for this function, ranging from minimally trained volunteers to paid paraprofessionals. Our work with PN's has contributed to the body of knowledge documenting that PNs can be effective in changing cancer health behaviors.[31,33,53]

3.8.2.b.TPN Intervention. The TPN intervention has been developed and tested by Dr. Paskett and her colleagues in a prior study and is well suited to translation to a rural population.[31] PN's will attempt to contact participants by telephone within 1 week after mailing the DVD. PNs will make at least 10 attempts at different times and on different days, including weekends, if needed. This timeframe will allow women to have received the DVD and watched it. If not viewed, the PN will encourage women to view the DVD and will assist to facilitate this. The PN will reinforce the DVD message and address barriers to receiving needed screening tests, including those not mentioned in the DVD. PNs will complete electronic encounter forms during each participant contact and have a file for each navigated participant. The encounter forms will include: 1) days/times of contact attempts; 2) a summary of the telephone call with documented cancer screening barriers; 3) navigator actions to address screening barriers; and 4) time spent on the call. PNs will track any other actions taken to assist participants (e.g. arrange transportation) and track the time taken to complete those actions. PN information will be directly submitted to a database that will alert them when a follow-up action needs to be placed for a participant (e.g. an alert will be sent to the PN to call the participant to remind them of a cancer screening appointment). PNs will make as many contacts as needed to assist participants to complete screening tests; however, a minimum of 2 calls will be placed to all participants randomized to PN.

3.8.2.c.PN Qualifications and Training. The success of a TPN intervention is dependent upon the navigator's ability to communicate with a wide range of people and in the culturally accepted vernacular. Personal skills such as the ability to be empathetic, patient, and caring are important when it comes to helping participants understand and comply with cancer screening tests. PNs should be familiar with the communities; therefore, they will be recruited from the geographic area where the study will occur. Navigators will be trained by Dr. Katz and Ms. Tatum, and will include information about cancer screening, treatment, and ways to overcome barriers to health care. Training of navigators will be held centrally (OH and IN together) and will include an explanation of the PN role as serving as a link between the participants and the health care system to guide participants to complete needed cancer screening tests. The following criteria will be assessed prior to allowing the PN to begin: 1) ability to communicate;2)knowledge/understanding of BC, CC, and CRC screening; and 3) understanding of the community and cultural setting (navigator materials; Appendix C). We will also have a Spanish-speaking PN.

3.8.2.d.Community Resources/Referral Network For The TPN Intervention. In Year 1, a listing of community resources needed to address barriers and needs of participants with no primary care provider, no regular source for screening tests, abnormal test results and cancer will be assembled by the project staff and PNs. We will involve local and state ACS offices as well as our State Agricultural Extension Offices located in each county. This listing will include medical resources, financial resources, transportation, local agencies, etc. For example, if a participant needs transportation, the PN will only query the data base for local transportation systems. Under the direction of Dr. Katz, low literacy educational resources will also be identified so that PNs can provide appropriate materials (e.g. from NCI, ACS, etc.). Most importantly, after outcome assessment, the PN will contact any women who received an abnormal screening test, regardless of study arm, to assure proper follow-up has occurred with her provider.

3.8.3. Intervention Pretesting. Usability testing will occur using both individual user feedback and focus groups. The CAB will be actively involved in development and evaluation of the TPN and TIDVD interventions to insure clarity, relevance and sensitivity. Board members will assist with recruitment of 20 rural women for focus groups and individual user testing. Usability will be evaluated by assessing ease of use, content (leveling and appropriateness), aesthetic appeal, and cultural relevance. We will present prototypes to members of the target population either individually or in group sessions as components are designed. The information gathered during individual user testing and focus group sessions will be used to revise both interventions.

3.9. Usual Care (UC). Women randomized to UC will receive brochures from the NCI (available also in Spanish) that explain and provide encouragement for BC, CC, and CRC screening. Participants will have a baseline survey prior to randomization that assesses beliefs, knowledge, self-reported CC, CRC, and BC screening, and other study variables. Women in UC group will complete T2 and T3 surveys as will women in other arms.

3.10. Recruitment of the Sample: Full Study

3.10.1. Sample Selection, Contact, and Consent. Eligible female residents of each of the 32 study counties will be randomly selected from a customized list provided by Survey Sampling International (commercial and United States Postal Service (USPS) lists) that includes female county residents age 50-74, inclusive (N=173,812). In order to ensure a sufficient number of truly rural women are enrolled, the sample will be stratified by Rural-Urban Commuting Area (RUCA) code which is assigned at the census tract level. An equal number of women will be sampled from tracts with RUCA codes of 4-6 and 7-10. Codes 4 through 6 represent micropolitan areas in and around towns with 10,000-50,000 residents where some residents may commute to a larger urban core. Codes 7 through 10 indicate small towns (population 2,500-9,999) and rural areas with little commuting to larger urban clusters. Mr. Young will provide selected names monthly to the OSU Behavioral Measurement Shared Resource (BMSR). We estimate that 55% of the women will be eligible (Section 3.5.1), 70% of eligible women will participate; thus to achieve our sample size of 1058, we will need to mail 2,748 letters.

Table 7. Measures

	Time(s) of Measurement*			
	1a	1b	2	3
Demographics	X			
General Health/Co-Morbidities	X			
Social Support	X			
Cancer History: Personal/Family	X			
Cancer Risk/Worry	X		X	X
Cancer Knowledge/Attitudes/Beliefs	X		X	X
Screening: Behaviors, Barriers, Benefits, Self-Efficacy	X		X	X
Staging	X		X	X
Screening Intention	X		X	X
Satisfaction with DVD (call in)		X		
Satisfaction with Navigator			X	
Contamination			X	X
County Level: Number of providers, screening facilities; median income level	X			
Medial Record Review	X			X

*Time(s) of measurement: 1a) Baseline; 1b) Post TIDVD; 2) 6 months; and 3) 12 months.

As in our previous studies in rural populations, selected women will be mailed a letter introducing the study, followed by a telephone call by BMSR trained interviewers 5 days after mailing the letter. The interviewers will receive a list of potential participants to contact by phone, and will make at least 10 attempts (calling at different times of the day, weekday, and weekends, as well as sending no contact letters) to reach the listed county resident. Once the participant is reached, the study will be explained, permission to verify eligibility will be obtained, the eligibility screener will be administered and if eligible, informed consent will be obtained (verbal consent with mailed consent form to follow). After verbal consent, the baseline survey will be administered. After completion of the baseline survey, participants will be mailed a medical release form with a $10 gift card, a stamped, pre-addressed envelope to mail the medical record release form to the OSU study office. Those who do not return the release form will be re-contacted by the interviewer. Staff will verify screening tests received by MRR and if the woman is still eligible, she will be randomized to one of 3 study arms. We have experience with these procedures from our ongoing studies (R24 MD002785, P50 CA015632).

The trained interviewers will be familiar with screening, determining eligibility, administering telephone surveys, and have a keen understanding of rural culture (Interviewer Training, Section 3.11.2.). We will also have a Spanish-speaking interviewer.

3.10.2. Randomization. Participants will be randomly assigned (2:2:1) to TIDVD alone, TIDVD + TPN, or UC. Randomization will be stratified by age (50-64 vs. 65+) and by the seven screening categories described in Table 5. A permuted-block randomization scheme will be used with blocks of size 2 and 4. A centralized web-based system at OSU will be used for all assignments. We are doing individual-level randomization vs. a group randomized trial (GRT) as the intervention is delivered to each woman at her home. We will assess for contamination at the 6 and 12 month surveys (Section 3.16).

3.11. Data Collection

3.11.1. Telephone Surveys. Trained interviewers from the BMSR will collect data via REDCap (Research Electronic Data Capture) at baseline, 6 and 12 months collecting information on outcomes as well as covariate and mediators. Self-report of cancer screening will be obtained at baseline, 6 and 12 months with MRR data collected at baseline and 12 months. Variables such as demographics that will not change are measured only at baseline. The 12 month survey will also include open-ended questions to assess why women are adherent to specific cancer screening tests and not adherent to other screening tests. Instruments are in Appendix D and constructs to be measured are described below and when each will be collected in Table 7.

Demographic/Medical History/Insurance Variables will assess age, race, education, income and marital status and have been used for descriptive reporting in our previous research without interpretation or scoring difficulties. We will also include questions on family history of cancer, insurance status, regular source of healthcare, health care professional's recommendations related to BC, CC, or CRC, and out-of-pocket cost for any BC, CC, and CRC screening.

Past Screening History will be measured by self-reported items that assess prior BC, CC, and CRC screening behaviors. Stage of adoption for BC, CC and CRC screening will be identified.[54,55] For example, items for CRC screening will determine whether participants: 1) have ever had CRC screening; 2) have thought about having CRC screening; 3) are planning (intend) to have a CRC screening test in the next 12 months; or 4) have an FOBT kit at home or a colonoscopy appointment.

Cancer Screening Knowledge and Beliefs. BC and CRC knowledge scales have been developed and tested by Drs. Champion and Rawl in preliminary studies.[57-60] A similar set of questions for CC has been used by Dr. Paskett. Several aspects of knowledge will be assessed, including risk factors, screening, and treatment. Belief scales for perceived risk, benefits, barriers, self-efficacy, and fear have been tested for validity and reliability for these screening tests. The same items will be used for beliefs scales for CC.

Perceived Risk. Perceived risk for BC and CRC include four items, a 3-item scale developed by Dr. Champion and a single-item measure designed to assess perceived age-adjusted risk.[57,58] We will add a scale to assess perceived risk of CC from Dr. Paskett's work. The items measure beliefs about the participant's perceived risk of getting each cancer in the future.

Benefits and Barriers of Cancer Screening. Dr. Champion has developed a "Benefits to Mammography" scale that assesses women's perceptions of mammography.[57] Benefits of FOBT and colonoscopy will be measured separately using summated Likert scales modified from those previously developed to measure BC screening benefits.[58,60] Barriers to mammography will be assessed using a 12-item scale developed by Dr. Champion.[57,59,60] Information on all items will be collected, although tailoring will be based on barriers found to be most relevant in prior work. Barriers to FOBT and colonoscopy will be measured separately using Likert scales modified by Dr. Rawl.[60] Items specific to CRC screening have been psychometrically tested in preliminary studies.[58] CC screening barrier questions from Dr. Paskett's work will be added.

Self-efficacy: Self-efficacy for mammography is measured on a 10-item scale developed and validated by Dr. Champion.[61] _Self-efficacy_ for FOBT and colonoscopy will be measured independently using 12-item with a Likert response.[26] In Year 1, we will refine a scale for CC screening.

Social Support/Social Network: The Medical Outcomes Study (MOS) social support instrument is easy to administer and provides four functional support scales (emotional/informational, tangible, affectionate, and positive social interaction) and an overall functional social support index.[62] In addition, each participant's social network will be documented regarding individuals who encourage/discourage cancer screening behaviors.

Community-Level Variables: Effect of community (neighborhood) factors, using geographic information system (GIS), on outcomes of receipt of needed screening tests will be evaluated. Participant addresses will be geocoded and accessibility to health care will be determined by measuring the number of facilities within a given distance of each geocoded address. Geocoded addresses will be spatially joined to a U.S. Census Topologically Integrated Geographic Encoding and Referencing (TIGER) zipcode shapefile containing demographic data. An area deprivation index score will be created using the health care accessibility variable and 21 U.S. Census data variables, including social and economic conditions (e.g. education, employment/occupation, housing conditions, income/ poverty, racial composition, residential stability).[63-66]

Participant satisfaction/engagement will be measured for the TIDVD and for the TPN. The participant engagement scale for TIDVD has 15 items that address interest, knowledge and engagement in the program and content. The items, piloted with 36 people, address ease of use, relevancy, information content, barriers, and general satisfaction. Satisfaction with TPN will be assessed with scales validated in the PNRP.[67]

3.11.2. Training of Interviewers. Interviewers will be trained, supervised, and monitored by the BMSR staff, in collaboration with the PIs**.** Training manuals for interviewers developed for prior studies will be modified for the proposed study and will include: 1) overview of study objectives; 2) description of interventions; 3) protection of human subjects, HIPPA, and confidentiality issues; 4) cultural sensitivity; 5) roles and responsibilities; 6) documentation and reporting requirements; 7) data monitoring and quality assurance procedures; 8) handling problems/questions during recruitment/data collection; 9) effective interviewing techniques; and 10) use of the REDCap telephone interview system. Following practice sessions, interviewers will role-play and receive feedback until they have reached 100% compliance with recruitment and data collection integrity.

3.11.3. Quality Assurance. Performance of interviewers will be closely monitored by the project manager and the BMSR supervisors. Approximately 25% of all interviews and recruitment calls will be monitored for quality assurance purposes. Feedback will be provided to the interviewers to correct performance weaknesses.

3.12. Medical Record Review (MRR)

Participants will be asked to sign and return a MRR form to the study office after the baseline interview, and the signed release will remain valid for the duration of the study. Dates of completed BC, CC, and CRC screening tests will be requested from health care providers named by participants during the baseline and 12-month interviews. The MRR form signed by the participant along with a request from our research office will be presented to the medical facility (secure fax, phone, or in person) where the cancer screening tests were ordered or performed. This request will inform the facility staff about the study and request information from the medical record about the participant's cancer screening history to be faxed or mailed to the study office at OSU. Non-responders will be contacted again, and if necessary, project staff will visit clinics to obtain this information. We anticipate that at least 85% of the participants will return a signed MRR form, and 95% of clinics will respond to our request for information (based on our prior studies).[52]

3.13. Statistical Analysis

3.13.1. Primary outcome. The primary outcome of adherence to screening guidelines at 12 months for Hypotheses 1 and 2 will combine MRR and self-report by using MRR when available and self-report otherwise. We anticipate obtaining MRR and verification on screening tests at the 12-month survey. Participants will be asked to provide contact information on both their primary care physician and any specialist who provided a screening test, reducing the chance of missing a test that was conducted but not reported to the primary care physician. For those who are lost to follow-up, a signed MRR form will enable us to obtain the adherence outcome for those participants. Thus, we anticipate adherence for the overwhelming majority of patients will be based on MRR. Dr. Monahan will supervise these analyses with assistance from Mr. Young.

For hypotheses 1 and 2, the differences in binary adherence (Hypothesis 1, all needed screenings; Hypothesis 2, any needed screening) across the three randomized arms will be tested initially with pair-wise chi-square tests. Binary logistic regression analysis will be used to compare the two interventions and the UC group on the binary dependent variable (adherence), while adjusting for any potentially confounding covariates. The models will be controlled for any demographic covariate (e.g., age, education) for which the randomized groups differ significantly at baseline using a liberal significance level of 0.20 to achieve conservative adjustment. As a sensitivity analysis, multiple imputation will be used to impute the adherence outcome for participants who are lost to follow-up and did not sign a MRR. We plan to include screening history as a covariate in our analysis by using the total number of past screenings for each test since the participant turned 50. All variables in the logistic regression models will be tested using the likelihood ratio test.[68] Adjusted odds ratios (ORs) and profile-likelihood-estimated 95% confidence intervals for those ORs will be provided. Our proposed theoretical model includes only variables that can be measured and used in modeling. The conceptual model (Figure 2) uses constructs from the Theory of Reasoned Action, and Theory of Planned Behavior, as well as other well-established behavioral theories.[39-42] Our model has taken these theories into consideration and added important constructs such as engagement. Therefore, we will perform theoretical modeling using structural equation modeling using the MPLUS software, as described in the next section. We have labeled this section as exploratory analyses because it is not part of the primary aims; however, the results will provide insightful findings about the context of the intervention effects including how all the theoretical variables interact with each other, both in terms of mediation and moderation effects.

3.13.2. Exploratory Aim. Associations between theoretical variables (community, social, and individual) and the binary screening outcomes will be identified with non-linear mixed models, accounting for two levels of assessment. The first level includes variables measured at the person level such as individual demographic and screening history variables, as well as beliefs which are measured for each participant in this study. The second level includes community variables measured at the county level such as number of providers, number of screening facilities, deprivation index, and median income level. Moderators of intervention effects on adherence will be identified by testing interaction terms in the model. The theoretical model in Figure 1, including mediation effects, will be tested with structural equation models using the MPLUS software.[69]

3.13.3. Evaluation of Cost-Effectiveness. Dr. Eric Seiber will assume responsibility for the cost-effectiveness analyses. The proposed economic evaluation of interventions can be seen as addressing three questions: 1) what are the full costs associated with the implementation of the interventions (cost-identification analysis); 2) what are the net costs associated with the interventions (is each intervention actually cost-saving?); and 3) what is the cost-effectiveness of each intervention?

3.13.3.a. Cost of the Interventions. The 1st question is answered by a careful accounting of the operational costs of the interventions, excluding those costs that are purely attributable to the research. Some of these are fixed costs associated with hiring and training PN's (which may recur if there is turnover in the position), and others are ongoing costs associated with the delivery of services. In the model implemented in this study, the PN's spend full time in that activity. Thus, the costs of implementation are straightforward and obtainable. Even though the PN is full-time, we will want to collect data concerning at least the broad categories of time use, so that we can estimate the cost of specific components (e.g., arranging transportation) more accurately. The best way to do this will be a structured time log. We will use a developed Participant Encounter Form and a Tracking Log of Direct Participant Contacts that will provide the majority of this information. The procedures outlined above will establish the aggregate cost of implementation. However, it will also be useful to measure cost on a per-unit basis. This will be done by calculating average cost per active participant per month and average cost per participant for cancer screening completion, for each test, type of test, and all needed tests.

3.13.3.b. Cost Savings. The 2nd question is whether each intervention is actually cost-saving, is essentially an attempt to calculate the numerator in a typical cost-effectiveness analysis:[70] the change in cost attributable to each intervention. Using the terminology of Gold et al., a full assessment would require measuring the changes in use of health care resources, changes in use of non-health care resources, changes in use of informal caregiver time, and changes in use of participant time for cancer screening.[70] In order to avoid distortions in the measured impact of an intervention due to pricing differences, all cancer screening tests will be assigned cost based on their Medicare allowable payment, regardless of how the service was actually paid for. Medicare payments are often used as a proxy for cost in cancer studies.[71,72] While this is not a pure cost measure, it is comparable and avoids other potential distortions like differences in cost to charge ratio for different payors.

3.13.3.c. Cost Effectiveness. The 3rd question, the cost-effectiveness of each intervention, will be the most difficult to address. Because of the nature of the interventions and the limited time for observation, it is not likely that the interventions will have a significant impact on standard measures of effectiveness used in cost-effectiveness analysis, such as quality-adjusted life years (QALYs). Cost-effectiveness analysis (CEA) implies a comparison of whether the cost per unit of outcome in one situation exceeds that in another. Unless the outcome measures are comparable, this is not possible. We will be able to examine outcomes of the interventions and the UC group (completed cancer screening tests). Our analysis will emphasize the first two questions (cost of the intervention itself, calculation of net cost). CEA will consist of measuring the incremental cost of achieving the observed incremental outcomes, but not a cost-effectiveness ratio in standard terms. Cost Utility: From our cost effectiveness estimates, we will conduct a QALY-based (Quality Adjusted Life Year) cost utility analysis. Calculating QALYs will require both utility (quality) weights and estimates of the life years saved. Both utility weights and the life years saved from the health outcomes will be drawn from the published literature. The Markov modeling will be conducted in TreeAge. Dr. Xu will conduct these analyses.

3.13.4. Process Evaluation. Dr. Mira Katz will conduct the process evaluation for the study using a mix of quantitative and qualitative methods focusing on the following 3 areas:

3.13.4.a. TIDVD Intervention. The process evaluation will include such key components as reasons for viewing/not viewing the TIDVD; participant satisfaction with the TIDVD and engagement with the intervention (Appendix D). The data collected on the 6 month survey will be used for this evaluation.

3.13.4.b. TPN Intervention. The TPN data collection form documents each participant's encounter(s) and lists the potential barriers and actions so the PN can easily document each participant-reported barrier and the associated PN actions. Analysis will be primarily descriptive but can address not only the frequencies of each barrier, but their clustering within individuals and distribution by geographic and demographic characteristics. Little is known about the range of barriers faced by rural women and the effective actions which can be taken to address cancer screening barriers. This data collection activity will provide rich descriptive data on these issues. Reports will include the number of participants reached with the content of the conversation, calls or contacts scheduled or rescheduled, and those who refused.

3.13.4.c. Quality Assurance for Intervention Fidelity. Evaluation of intervention processes is needed to ensure consistency of intervention delivery. Modifications will be made as necessary and recorded to ensure appropriate intervention delivery and maintenance of protocol integrity. Evaluation questions will assess user experience/satisfaction with the intervention (Appendix D). A random sample of 10 participants per month will be called by the project manager to confirm receipt of the DVD/PN calls. Since all intervention participants will receive the DVD, we will include a message at the end of the program informing them of the opportunity to call a toll-free number to obtain a $10 gift card in appreciation for their time. This incentive will serve as a rough manipulation check allowing us to document whether participants viewed the entire video. Participants who call

in will be offered an additional $10 to complete a brief interview to assess their experience and satisfaction with the DVD. The BMSR will provide quality checks on 10% of the TPN calls to document that the telephone call was made, if cancer screening barriers were reported, and the actions discussed match the TPN document.

3.14. Management Plan

Overall study responsibility belongs to Drs. Paskett and Champion, Co-PIs. Dr. Paskett and her staff will be responsible for day-to-day study oversight including obtaining human subjects approval, drawing/recruiting the sample, conducting interviews and the TPN intervention, and data management activities. Dr. Champion and her staff will be responsible for the refining and troubleshooting the TIDVD, and supervising the data analyses. Dr. Katz will assist in training the PNs and overseeing the process evaluation. Dr. Rawl will assist with refinement of the DVD program and study measures. Dr. Grever will serve as the team clinician to advise about medical-related issues and to provide expertise related to clinical environments which serve these women. Dr. Seiber will lead the cost analysis with assistance from Dr. Xu, and Dr. Monahan will lead data analysis with assistance from Mr. Young.

3.15. Timeline (Table 8)

3.16. Potential Problems and Strategies

While there is potential to produce important information, there are limitations and possible problems to consider. Several are listed with plans to minimize or address each potential limitation or problem. ***Loss to follow-up:*** It is anticipated that there will be some loss to follow-up because of participant dropout, death, or relocation. Based on our preliminary studies, we estimate loss to be no more than 10% over the 12-month period, which was accounted for in the power analysis and sample size determination. ***Missing data:*** Missing data will be minimized through the use of telephone data collection methods and through interviewer training. All characteristics at baseline will be examined to determine potential bias that would require subsequent caution regarding interpretation of results. ***Limited internal validity:*** Randomization will minimize the influence of extraneous variables on outcomes. ***Sample bias:*** Based on preliminary studies, we estimate that approximately 70% of eligible women who are contacted will participate. It is possible that those who are motivated to complete screening tests will self-select into the study. To determine the representativeness of our sample, we will compare demographic characteristics of those who agree to participate with those who do not from data collected on the screening form. ***Outcome Ascertainment:*** Limitations of self-report for completion of cancer screening tests are well documented. Therefore, our outcomes, cancer screening rates, will be calculated from medical record reports.

Table 8. Timeline (months)

**Year 1**	**1**	**2**	**3**	**4**	**5**	**6**	**7**	**8**	**9**	**10**	**11**	**12**
Start-up activities/Hire and train staff	•	•	•	•	•	•	•	•	•	•	•	•
Refinement of materials					•	•	•	•	•	•	•	•
Community advisory board meetings	•							•				
Monthly team meetings	•	•	•	•	•	•	•	•	•	•	•	•
**Year 2**	**13**	**14**	**15**	**16**	**17**	**18**	**19**	**20**	**21**	**22**	**23**	**24**
Recruitment of women/Intervention delivery	•	•	•	•	•	•	•	•	•	•	•	•
Surveys: baseline, 6 months, and MRR	•	•	•	•	•	•	•	•	•	•	•	•
Data cleaning and management	•	•	•	•	•	•	•	•	•	•	•	•
Community advisory board meetings	•							•				
Monthly team meetings	•	•	•	•	•	•	•	•	•	•	•	•
**Year 3**	**25**	**26**	**27**	**28**	**29**	**30**	**31**	**32**	**33**	**34**	**35**	**36**
Recruitment of women/Intervention delivery	•	•	•	•	•	•	•	•	•	•	•	•
Surveys: baseline, 6, 12 months, and MRR	•	•	•	•	•	•	•	•	•	•	•	•
Data cleaning and management	•	•	•	•	•	•	•	•	•	•	•	•
Community advisory board meetings	•							•				
Monthly team meetings	•	•	•	•	•	•	•	•	•	•	•	•
**Year 4**	**37**	**38**	**39**	**40**	**41**	**42**	**43**	**44**	**45**	**46**	**47**	**48**
Recruitment of women/Intervention delivery	•	•	•	•	•	•	•	•	•	•	•	•
Surveys: baseline, 6, 12 months, and MRR	•	•	•	•	•	•	•	•	•	•	•	•
Data cleaning and management	•	•	•	•	•	•	•	•	•	•	•	•
Community advisory board meetings	•							•				
Monthly team meetings	•	•	•	•	•	•	•	•	•	•	•	•
**Year 5**	**49**	**50**	**51**	**52**	**53**	**54**	**55**	**56**	**57**	**58**	**59**	**60**
Intervention delivery	•	•	•	•								
Surveys: 12 months and MRR	•	•	•	•	•							
Data cleaning and management	•	•	•	•	•							
Community advisory board meetings	•							•				
Report writing/grant submission/project shut down						•	•	•	•	•	•	•
Monthly team meetings	•	•	•	•	•	•	•	•	•	•	•	•

Contamination: In any intervention study, contamination of the control participants is always a concern, especially with individual vs. a GRT. The TIDVD is mailed to the woman's home and the PN intervention is telephone-based, so contamination should be minimized since the intervention is delivered to each woman. In

addition, as in our prior studies, we will assess for contamination in the 6 and 12 month surveys. We have not found a high degree of contamination in previous studies and since this is a rural setting, interactions among women are more limited than when interventions are delivered in other settings, e.g. churches or clinics, where women may interact.

REFERENCES

1. SEER Cancer Statistics Review, 1975-2010. 2013. (Accessed at http://seer.cancer.gov/csr/1975_2010.)
2. American Cancer Society. Cancer Facts and Figures 2014. Atlanta; American Cancer Society; 2014.
3. American Cancer Society. Cancer Prevention and Early Detection Facts and Figures 2013. Atlanta; American Cancer Society; 2013.
4. Klabunde CN, Cronin KA, Breen N, Waldron WR, Ambs AH, Nadel MR. Trends in colorectal cancer test use among vulnerable populations in the United States. Cancer Epidemiol Biomarkers Prev 2011;20:1611-21.
5. Swan J, Breen N, Graubard BI, et al. Data and trends in cancer screening in the United States: results from the 2005 National Health Interview Survey. Cancer 2010;116:4872-81.
6. Singh GK. Rural-urban trends and patterns in cervical cancer mortality, incidence, stage, and survival in the United States, 1950-2008. J Community Health 2012;37:217-23.
7. Paskett ED, Fisher JL, Lengerich EJ, et al. Disparities in underserved white populations: the case of cancer-related disparities in Appalachia. Oncologist 2011;16:1072-81.
8. Miller MK, Stokes CS, Clifford WB. A comparison of the rural-urban mortality differential for deaths from all causes, cardiovascular disease and cancer. J Rural Health 1987;3:23-34.
9. Monroe AC, Ricketts TC, Savitz LA. Cancer in rural versus urban populations: a review. J Rural Health 1992;8:212-20.
10. Campbell MK, James A, Hudson MA, et al. Improving multiple behaviors for colorectal cancer prevention among African American church members. Health Psychol 2004;23:492-502.
11. Meissner HI, Yabroff KR, Dodd KW, Leader AE, Ballard-Barbash R, Berrigan D. Are patterns of health behavior associated with cancer screening? Am J Health Promot 2009;23:168-75.

12. Prochaska JO. Multiple Health Behavior Research represents the future of preventive medicine. Prev Med 2008;46:281-5.
13. Meissner HI, Vernon SW, Rimer BK, et al. The future of research that promotes cancer screening. Cancer 2004;101:1251-9.
14. Hartley D. Rural health disparities, population health, and rural culture. Am J Public Health 2004;94: 1675-8.
15. Jones CA PT, Ahearn M, Mishra AK, Variyam JN. Health Status and Health Care Access of Farm and Rural Populations; 2009.
16. Liff JM, Chow WH, Greenberg RS. Rural-urban differences in stage at diagnosis. Possible relationship to cancer screening. Cancer 1991;67:1454-9.
17. National Center for Health Statistics. United States, 2011: With Special Feature on Socioeconomic Status and Health. Hyattsville, MD: National Center for Health Statistics; 2012.
18. Smith RA, Cokkinides V, Brooks D, Saslow D, Shah M, Brawley OW. Cancer screening in the United States, 2011: A review of current American Cancer Society guidelines and issues in cancer screening. CA Cancer J Clin 2011;61:8-30.
19. Paskett E, Tatum C, Rushing J, et al. Randomized trial of an intervention to improve mammography utilization among a triracial rural population of women. J Natl Cancer Inst 2006;98:1226-37.
20. Paskett ED, McLaughlin JM, Lehman AM, Katz ML, Tatum CM, Oliveri JM. Evaluating the efficacy of lay health advisors for increasing risk-appropriate Pap test screening: a randomized controlled trial among Ohio Appalachian women. Cancer Epidemiol Biomarkers Prev 2011;20:835-43.
21. Paskett ED, Tatum C, Rushing J, et al. Racial differences in knowledge, attitudes, and cancer screening practices among a triracial rural population. Cancer 2004;101:2650-9.
22. Paskett ED, Tatum CM, D'Agostino R, Jr., et al. Community-based interventions to improve breast and cervical cancer screening: results of the Forsyth County Cancer Screening (FoCaS) Project. Cancer Epidemiol Biomarkers Prev 1999;8:453-9.
23. Katz ML, Fisher JL, Fleming K, Paskett ED. Patient activation increases colorectal cancer screening rates: a randomized trial among low-income minority patients. Cancer Epidemiol Biomarkers Prev;21:45-52.
24. Champion V, Skinner CS, Hui S, et al. The effect of telephone versus print tailoring for mammography adherence. Patient Educ Couns 2007;65:416-23.
25. Champion VS, J; Hui, S; Saywell, RM, Jr.; Zollinger, TW; Ray, D. Community intervention to increase mammography screening. In Bethesda, MD: National Cancer Institute; 1998-2002.
26. Menon U. Factors associated with colorectal cancer screening in an average risk population [Dissertation]. Indianapolis, IN: Indiana University; 2000.
27. Rawl SM, Champion VL, Scott LL, et al. A randomized trial of two print interventions to increase colon cancer screening among first-degree relatives. Patient Educ Couns 2008;71:215-27.
28. Rawl SM, Skinner CS, Perkins SM, et al. Computer-delivered tailored intervention improves colon cancer screening knowledge and health beliefs of African-Americans. Health Educ Res. 2012;27:868-885.
29. Saslow D, Solomon D, Lawson HW, et al. American Cancer Society, American Society for Colposcopy and Cervical Pathology, and American Society for Clinical Pathology screening guidelines for the prevention and early detection of cervical cancer. CA Cancer J Clin 2012;62:147-72.
30. Screening for breast cancer: U.S. Preventive Services Task Force recommendation statement. Ann Intern Med 2009;151:716-26, W-236.
31. Paskett ED, Katz ML, Post DM, Pennell ML, Young GS, Seiber EE, Harrop JP, DeGraffinreid CR, Tatum CM, Dean JA, Murray DM. The Ohio Patient Navigation Research Program: Does the American Cancer Society navigation model improve time to resolution among patients with abnormal screening tests? Cancer Epidemiology Biomarkers & Prevention 2012;21:1620-1628.
32. Russell KM, Champion VL, Monahan PO, et al. Randomized trial of a lay health advisor and computer intervention to increase mammography screening in African American women. Cancer Epidemiol Biomarkers Prev 2010;19:201-10.
33. Freund KM, et al. Impact of patient navigation on timely cancer care: the Patient Navigation Research Program. J Natl Cancer Inst. 2014;106:p. dju115.
34. Warnecke RB, Oh A, Breen N, et al. Approaching health disparities from a population perspective: the National Institutes of Health Centers for Population Health and Health Disparities. Am J Public Health 2008;98:1608-15.
35. Noar SM, Chabot M, Zimmerman RS. Applying health behavior theory to multiple behavior change: considerations and approaches. Prev Med 2008;46:275-80.

36. Prochaska JJ, Spring B, Nigg CR. Multiple health behavior change research: an introduction and overview. Prev Med 2008;46:181-8.
37. Norcross JC, Krebs PM, Prochaska JO. Stages of change. J Clin Psychol;67:143-54.
38. Rosenstock IM, Strecher VJ, Becker MH. Social learning theory and the Health Belief Model. Health Educ Q 1988;15:175-83.
39. Prochaska JO, Redding CA, Evers KE. The Transtheoretical Model and Stages of Change. In: Glanz K, Rimer BK, Viswanath K, Eds. Health Behavior and Health Education Theory, Research, and Practice. San Francisco, CA: Jossey-Bass. John Wiley & Sons, Inc/; 2008:97-122.
40. Montano DE, Kasprzyk, D. Theory of reasoned action, theory of planned behavior, and the integrated behavioral model. In: Glanz K RB, Viswanath K, ed. Health Behavior and Health Education Theory, Research, and Practice. 4th ed. San Francisco: Jossey-Bass; 2008:67-96.
41. Ajzen I. The Theory of Planned Behavior. Organizational Behavior and Human Decision Processes 1991;50:179-211.
42. Janz NK, Becker MH. The Health Belief Model: a decade later. Health Educ Q 1984;11:1-47.
43. Becker MH. Health Belief Model and Personal Health Behavior. San Francisco: Society for Public Health Education; 1974.
44. Stockwell DH, Woo P, Jacobson BC, et al. Determinants of colorectal cancer screening in women undergoing mammography. Am J Gastroenterol 2003;98:1875-80.
45. Coulter A, Ellins J. Effectiveness of strategies for informing, educating, and involving patients. BMJ 2007; 335: 24-27.
46. Katz ML, Tatum C, Dickinson SL, et al. Improving colorectal cancer screening by using community volunteers: results of the Carolinas cancer education and screening (CARES) project. Cancer 2007;110:1602-10.
47. Champion VL, Springston JK, Zollinger TW, et al. Comparison of three interventions to increase mammography screening in low income African American women. Cancer Detect Prev 2006;30:535-44.
48. Skinner CS, Buchanan A, Champion V, et al. Process outcomes from a randomized controlled trial comparing tailored mammography interventions delivered via telephone vs. DVD. Patient Educ Couns 2011;85:308-12.
49. Champion VL, Rawl SM, Bourff SA, Champion KM, Smith LG, Buchanan AH, et al. Randomized Trial of DVD, Telephone, and Usual Care for Increasing Mammography Adherence. Journal of Health Psychology. 2014, Jul 28 Epub. PMID: 25070967
50. Russell KM. Combined mammography interventions for Black Women: Indiana University; National Institutes of Health; 2005.
51. American Cancer Society. Cancer Facts and Figures 2012. Atlanta: American Cancer Society; 2012.
52. Llanos AA, Pennell ML, Young GS, Tatum CM, Katz ML, Paskett ED. No association between colorectal cancer worry and screening uptake in Appalachian Ohio. J Public Health 2014 May 20 Epub
53. Paskett ED, Harrop JP, Wells KJ. Patient navigation: an update on the state of the science. CA Cancer J Clin 2011;61:237-49.
54. Rakowski W, Dube CE, Marcus BH, Prochaska JO, Velicer WF, Abrams DB. Assessing elements of women's decisions about mammography. Health Psychol 1992;11:111-8.
55. Rakowski W, Fulton JP, Feldman JP. Women's decision making about mammography: a replication of the relationship between stages of adoption and decisional balance. Health Psychol 1993;12:209-14.
56. Noar SM, Benac CN, Harris MS. Does tailoring matter? Meta-analytic review of tailored print health behavior change interventions. Psychol Bull 2007;133:673-93.
57. Champion VL. Revised susceptibility, benefits, and barriers scale for mammography screening. Res Nurs Health 1999;22:341-8.
58. Rawl SC, Champion V, Menon U, Loehrer PJ, Vance GH, Skinner CS. Validation of Scales to Measure Benefits of and Barriers to Colorectal Cancer Screening. Journal of Psychosocial Oncology 2001;19: 47-63.
59. Champion VL. Instrument development for health belief model constructs. Advances in Nursing Science 1984;6:73-85.
60. Champion VL. Instrument refinement for breast cancer screening behaviors. Nursing Research 1993; 42: 139-43.
61. Champion VL, Skinner CS, Menon U. Development of a self-efficacy scale for mammography. Research in Nursing and Health 2005;28:329-36.
62. Sherbourne CD, Stewart AL. The MOS social support survey. Soc Sci Med 1991;32:705-14.

63. Singh GK. Area deprivation and widening inequalities in US mortality, 1969-1998. Am J Public Health 2003;93:1137-43.
64. Messer LC, Laraia BA, Kaufman JS, et al. The development of a standardized neighborhood deprivation index. J Urban Health 2006;83:1041-62.
65. Krieger N, Chen JT, Waterman PD, Soobader MJ, Subramanian SV, Carson R. Geocoding and monitoring of US socioeconomic inequalities in mortality and cancer incidence: does the choice of area-based measure and geographic level matter?: the Public Health Disparities Geocoding Project. Am J Epidemiol 2002; 156:471-82.
66. Diez-Roux AV, Kiefe CI, Jacobs DR, Jr., et al. Area characteristics and individual-level socioeconomic position indicators in three population-based epidemiologic studies. Ann Epidemiol 2001;11:395-405.
67. Jean-Pierre P, Fiscella K, Winters PC, Paskett E, Wells K, Battaglia T. Psychometric validation and reliability analysis of a Spanish version of the patient satisfaction with cancer-related care measure: a patient navigation research program study. Support Care Cancer;20:1949-56.
68. Hosmer DW, Lemeshow S. Applied logistic regression. 2nd ed. New York: Wiley; 2000.
69. Muthen LK, Muthen, B.O. Mplus User's Guide. Los Angeles, CA; 1998-2010.
70. Gold MR, Siegel, J.E., Russell, L.B., Weinstein, M.C. Cost-Effectiveness in Health and Medicine. New York: Oxford University Press; 1996.
71. Hayman JA, Langa KM, Kabeto MU, et al. Estimating the cost of informal caregiving for elderly patients with cancer. J Clin Oncol 2001;19:3219-25.
72. Sherman EJ, Pfister DG, Ruchlin HS, et al. The Collection of Indirect and Nonmedical Direct Costs (COIN) form: a new tool for collecting the invisible costs of androgen independent prostate carcinoma. Cancer 2001;91:841-53.
73. Katz ML, Reiter PI, Young GS, Pennell ML, Tatum CM, Paskett ED. Adherence to multiple cancer screening tests among women living Appalachia Ohio. In review
74. Emmons KM, McBride CM, Puleo E, Pollak KI, Clipp E, Kuntz K, et al. Project PREVENT: A randomized trial to reduce multiple behavioral risk factors for colon cancer. Cancer Epidemiol Biomarkers Prev 2005;14:1453-1459.
75. Lynch BM, Couneya KS, Sethi P, Patrao TA, Hawkes AL. A randomized controlled trial of a multiple health behavior change intervention delivered to colorectal cancer survivors. Cancer 2014; 120:2665-2672.
76. Johnson SS, Pavia AL, Mauriello L, Prochaska JO, Redding C, Velicer WF. Coaction in multiple behavior change interventions: Consistency across multiple studies on weight management and obesity prevention. Health Psychology 2014;33:475-480.
