# Supplementary material for: Comparative Effectiveness of 2 Interventions to Increase Breast, Cervical, and Colorectal Cancer Screening Among Women in the Rural US: A Randomized Clinical Trial
Source: JAMA Netw Open. 2023 Apr 28;6(4):e2311004. doi: 10.1001/jamanetworkopen.2023.11004 (PMC10148202; doi:10.1001/jamanetworkopen.2023.11004)
Supplement: Supplement 2. — eTable 1. Bivariate Analysis of Baseline Covariates and Screening Outcomes eTable 2. Logistic Regression for All Cancer Screenings at 12 Months Postbaseline (n = 852) eTable 3. Logistic Regression for Any Cancer Screening at 12 Months Postbaseline (n = 847) eAppendix. Intervention Cost Methods [file jamanetwopen-e2311004-s002.pdf]

Supplemental Online Content

Champion VL, Paskett ED, Stump TE, et al. Comparative effectiveness of 2 interventions to increase breast, cervical, and colorectal cancer screening among women in the rural US. *JAMA Netw Open*. 2023;6(4):e2311004. doi:10.1001/jamanetworkopen.2023.11004

eTable 1. Bivariate Analysis of Baseline Covariates and Screening Outcomes

eTable 2. Logistic Regression for All Cancer Screenings at 12 Months Postbaseline (n = 852)

eTable 3. Logistic Regression for Any Cancer Screening at 12 Months Postbaseline (n = 847)

eAppendix. Intervention Cost Methods

This supplemental material has been provided by the authors to give readers additional information about their work.

© 2023 Champion VL et al. *JAMA Network Open.*

eTable 1. Bivariate Analysis of Baseline Covariates and Screening Outcomes

	UTD for All cancer screenings			UTD for Any cancer screenings		
	No record of test or outside window (N=769)	Received within 12mo window (N=194)	p value	No record of test or outside window (N=616)	Received within 12mo window (N=347)	p value
**Study design characteristics**						
Randomized arm:			< 0.001[b]			< 0.001[b]
Usual care	174 (90.2%)	19 (9.8%)		145 (75.1%)	48 (24.9%)	
DVD	325 (85.1%)	57 (14.9%)		272 (71.2%)	110 (28.8%)	
DVD/Navigator	270 (69.6%)	118 (30.4%)		199 (51.3%)	189 (48.7%)	
Baseline Breast cancer screening status:			0.061[a]			< 0.001[a]
Within screening guidelines (UTD)	436 (77.7%)	125 (22.3%)		394 (70.2%)	167 (29.8%)	
Outside screening guidelines (not UTD)	333 (82.8%)	69 (17.2%)		222 (55.2%)	180 (44.8%)	
Baseline Colorectal cancer screening status:			0.015[a]			0.111[a]
Within screening guidelines (UTD)	225 (75.0%)	75 (25.0%)		203 (67.7%)	97 (32.3%)	
Outside screening guidelines (not UTD)	544 (82.1%)	119 (17.9%)		413 (62.3%)	250 (37.7%)	
Baseline Cervical cancer screening status:			< 0.001[a]			0.278[a]
Within screening guidelines (UTD)	279 (68.0%)	131 (32.0%)		254 (62.0%)	156 (38.0%)	
Outside screening guidelines (not UTD)	490 (88.6%)	63 (11.4%)		362 (65.5%)	191 (34.5%)	
Baseline screening status combinations (not UTD):			< 0.001[b]			< 0.001[b]

© 2023 Champion VL et al. *JAMA Network Open.*

Breast, colorectal, and cervical	175 (94.1%)	11 (5.9%)		111 (59.7%)	75 (40.3%)	
Breast and colorectal	74 (83.1%)	15 (16.9%)		49 (55.1%)	40 (44.9%)	
	UTD for All cancer screenings			UTD for Any cancer screenings		
	No record of test or outside window (N=769)	Received within 12mo window (N=194)	p value	No record of test or outside window (N=616)	Received within 12mo window (N=347)	p value
Breast and cervical	58 (85.3%)	10 (14.7%)		36 (52.9%)	32 (47.1%)	
Colorectal and cervical	116 (92.1%)	10 (7.9%)		74 (58.7%)	52 (41.3%)	
Breast only	26 (44.1%)	33 (55.9%)		26 (44.1%)	33 (55.9%)	
Colorectal only	179 (68.3%)	83 (31.7%)		179 (68.3%)	83 (31.7%)	
Cervical only	141 (81.5%)	32 (18.5%)		141 (81.5%)	32 (18.5%)	
Baseline screening status combinations (not UTD) re-calculated for sensitivity analysis, where all participants age 66+ coded as UTD at baseline for cervical:			< 0.001[b]			< 0.001[b]
Mammography, CRC and PAP	141 (93.4%)	10 (6.6%)		91 (60.3%)	60 (39.7%)	
Mammography and CRC	108 (87.1%)	16 (12.9%)		69 (55.6%)	55 (44.4%)	
Mammography and PAP	45 (83.3%)	9 (16.7%)		27 (50.0%)	27 (50.0%)	
CRC and PAP	84 (90.3%)	9 (9.7%)		52 (55.9%)	41 (44.1%)	
Mammography only	39 (53.4%)	34 (46.6%)		35 (47.9%)	38 (52.1%)	
CRC only	211 (71.5%)	84 (28.5%)		201 (68.1%)	94 (31.9%)	
PAP only	86 (75.4%)	28 (24.6%)		86 (75.4%)	28 (24.6%)	
N-Miss	55	4		55	4	
Medical record location confirmed vs not confirmed at 12 months[d]			< 0.001[a]			< 0.001[a]

© 2023 Champion VL et al. *JAMA Network Open.*

	UTD for All cancer screenings			UTD for Any cancer screenings		
	No record of test or outside window (N=769)	Received within 12mo window (N=194)	p value	No record of test or outside window (N=616)	Received within 12mo window (N=347)	p value
Confirmed	659 (78.1%)	185 (21.9%)		519 (61.5%)	325 (38.5%)	
Not confirmed	110 (92.4%)	9 (7.6%)		97 (81.5%)	22 (18.5%)	
State:			0.622[a]			0.731[a]
Indiana	305 (80.7%)	73 (19.3%)		239 (63.2%)	139 (36.8%)	
Ohio	464 (79.3%)	121 (20.7%)		377 (64.4%)	208 (35.6%)	
**Participant Demographics**						
Age at consent			0.004[c]			0.002[c]
Mean (SD)	58.9 (6.5)	57.4 (5.6)		59.0 (6.4)	57.7 (6.1)	
Median (Q1, Q3)	58.0 (53.0, 63.0)	57.0 (52.2, 61.8)		59.0 (53.0, 64.0)	57.0 (52.0, 62.0)	
Range	50.0 - 74.0	50.0 - 72.0		50.0 - 74.0	50.0 - 74.0	
Age-3 categories			0.013[b]			0.022[b]
<65	596 (78.0%)	168 (22.0%)		472 (61.8%)	292 (38.2%)	
65-69	118 (85.5%)	20 (14.5%)		99 (71.7%)	39 (28.3%)	
70+	55 (90.2%)	6 (9.8%)		45 (73.8%)	16 (26.2%)	
Age-4 categories			0.033[b]			0.012[b]
50-54	253 (77.4%)	74 (22.6%)		199 (60.9%)	128 (39.1%)	
55-59	185 (77.1%)	55 (22.9%)		141 (58.8%)	99 (41.2%)	
60-64	158 (80.2%)	39 (19.8%)		132 (67.0%)	65 (33.0%)	

© 2023 Champion VL et al. *JAMA Network Open.*

	UTD for All cancer screenings			UTD for Any cancer screenings		
	No record of test or outside window (N=769)	Received within 12mo window (N=194)	p value	No record of test or outside window (N=616)	Received within 12mo window (N=347)	p value
65+	173 (86.9%)	26 (13.1%)		144 (72.4%)	55 (27.6%)	
Education			< 0.001[b]			0.007[b]
HS/GED or less	131 (87.3%)	19 (12.7%)		110 (73.3%)	40 (26.7%)	
Some college or Associate Degree	303 (82.6%)	64 (17.4%)		243 (66.2%)	124 (33.8%)	
BS/BA/AB/BSN	198 (79.2%)	52 (20.8%)		151 (60.4%)	99 (39.6%)	
MS or more	137 (69.9%)	59 (30.1%)		112 (57.1%)	84 (42.9%)	
Income			0.005[b]			0.195[b]
<$40k	155 (86.6%)	24 (13.4%)		124 (69.3%)	55 (30.7%)	
$40k - $79,999	289 (82.3%)	62 (17.7%)		228 (65.0%)	123 (35.0%)	
$80k+	296 (74.7%)	100 (25.3%)		239 (60.4%)	157 (39.6%)	
Unknown	29 (78.4%)	8 (21.6%)		25 (67.6%)	12 (32.4%)	
How would you describe your household's financial situation right now?			0.044[b]			0.016[b]
After paying the bills, you still have enough money for special things that you want.	457 (77.7%)	131 (22.3%)		362 (61.6%)	226 (38.4%)	
You have enough money to pay the bills, but little extra money to buy something special that you want.	218 (81.3%)	50 (18.7%)		173 (64.6%)	95 (35.4%)	
You have money to pay the bills only by cutting back or are having difficulty paying the bills.	88 (88.0%)	12 (12.0%)		76 (76.0%)	24 (24.0%)	
N-Miss	6	1		5	2	
Marital status			0.477[b]			0.257[b]

© 2023 Champion VL et al. *JAMA Network Open.*

Married/living as married	589 (79.3%)	154 (20.7%)		475 (63.9%)	268 (36.1%)	
Divorced/Widowed/Separated	148 (80.4%)	36 (19.6%)		113 (61.4%)	71 (38.6%)	
	UTD for All cancer screenings			UTD for Any cancer screenings		
	No record of test or outside window (N=769)	Received within 12mo window (N=194)	p value	No record of test or outside window (N=616)	Received within 12mo window (N=347)	p value
Never married	30 (88.2%)	4 (11.8%)		26 (76.5%)	8 (23.5%)	
N-Miss	2	0		2	0	
Insurance			0.004[b]			0.005[b]
No insurance	45 (91.8%)	4 (8.2%)		32 (65.3%)	17 (34.7%)	
Public only	73 (85.9%)	12 (14.1%)		66 (77.6%)	19 (22.4%)	
Private only	514 (76.8%)	155 (23.2%)		406 (60.7%)	263 (39.3%)	
Public and private	136 (85.5%)	23 (14.5%)		111 (69.8%)	48 (30.2%)	
N-Miss	1	0		1	0	
Race			0.808[a]			0.841[a]
Non-White	21 (77.8%)	6 (22.2%)		18 (66.7%)	9 (33.3%)	
White	748 (79.9%)	188 (20.1%)		598 (63.9%)	338 (36.1%)	
Do you consider yourself of Hispanic, Latino, or Spanish origin?			0.100[a]			0.673[a]
No	766 (80.0%)	191 (20.0%)		613 (64.1%)	344 (35.9%)	
Yes	3 (50.0%)	3 (50.0%)		3 (50.0%)	3 (50.0%)	
Are you currently working for pay?			< 0.001[b]			< 0.001[b]
No	267 (85.0%)	47 (15.0%)		229 (72.9%)	85 (27.1%)	
Yes - part time	155 (83.3%)	31 (16.7%)		120 (64.5%)	66 (35.5%)	

© 2023 Champion VL et al. *JAMA Network Open.*

	UTD for All cancer screenings			UTD for Any cancer screenings		
Yes - full time	347 (74.9%)	116 (25.1%)		267 (57.7%)	196 (42.3%)	
	No record of test or outside window (N=769)	Received within 12mo window (N=194)	p value	No record of test or outside window (N=616)	Received within 12mo window (N=347)	p value
Mother's education			0.918[b]			0.935[b]
HS/GED or less	434 (80.2%)	107 (19.8%)		352 (65.1%)	189 (34.9%)	
Some college or AS	176 (80.7%)	42 (19.3%)		137 (62.8%)	81 (37.2%)	
BS/BA/AB/BSN	91 (78.4%)	25 (21.6%)		73 (62.9%)	43 (37.1%)	
MS or more	48 (76.2%)	15 (23.8%)		39 (61.9%)	24 (38.1%)	
Unknown	20 (80.0%)	5 (20.0%)		15 (60.0%)	10 (40.0%)	
Where would you place yourself on this ladder (community)?			0.009[c]			0.848[c]
Mean (SD)	6.6 (1.7)	6.9 (1.6)		6.6 (1.7)	6.6 (1.6)	
Median (Q1, Q3)	7.0 (6.0, 8.0)	7.0 (6.0, 8.0)		7.0 (6.0, 8.0)	7.0 (6.0, 8.0)	
Range	1.0 - 10.0	2.0 - 10.0		1.0 - 10.0	1.0 - 10.0	
N-Miss	11	4		9	6	
Where would you place yourself on this ladder (US)?			0.010[c]			0.203[c]
Mean (SD)	6.1 (1.6)	6.4 (1.5)		6.1 (1.7)	6.2 (1.5)	
Median (Q1, Q3)	6.0 (5.0, 7.0)	7.0 (6.0, 7.0)		6.0 (5.0, 7.0)	6.0 (5.0, 7.0)	
Range	1.0 - 10.0	2.0 - 10.0		1.0 - 10.0	2.0 - 10.0	
N-Miss	12	5		9	8	
U.S.-based percentile of block group Area Deprivation Index (ADI) score			0,004[c]			0.031[c]

© 2023 Champion VL et al. *JAMA Network Open.*

	UTD for All cancer screenings			UTD for Any cancer screenings		
	No record of test or outside window (N=769)	Received within 12mo window (N=194)	p value	No record of test or outside window (N=616)	Received within 12mo window (N=347)	p value
Mean (SD)	67.3 (15.9)	63.5 (15.8)		67.4 (16.1)	65.0 (15.6)	
Median (Q1, Q3)	68.0 (55.0, 80.0)	63.0 (51.0, 76.0)		68.0 (55.0, 80.0)	66.0 (53.0, 77.0)	
Range	9.0 - 100.0	21.0 - 99.0		9.0 - 100.0	21.0 - 99.0	
N-Miss	43	13		32	24	
Secondary RUCA Code–recoded, categorization B–2 categories			0.065[b]			0.080[b]
Urban and Large Rural City/Town	507 (81.6%)	114 (18.4%)		410 (66.0%)	211 (34.0%)	
Small and Isolated Small Rural Town	262 (76.6%)	80 (23.4%)		206 (60.2%)	136 (39.8%)	
U.S.-based quintile of Yost Index			0.595[b]			0.410[b]
1st quintile (Lowest SES)	124 (83.2%)	25 (16.8%)		104 (69.8%)	45 (30.2%)	
2nd quintile	264 (78.8%)	71 (21.2%)		205 (61.2%)	130 (38.8%)	
3rd quintile	240 (80.3%)	59 (19.7%)		192 (64.2%)	107 (35.8%)	
4th quintile	71 (75.5%)	23 (24.5%)		62 (66.0%)	32 (34.0%)	
5th quintile (Highest SES)	1 (100.0%)	0 (0.0%)		1 (100.0%)	0 (0.0%)	
N-Miss	69	16		52	33	
**Health behaviors and characteristics**						
Smoking status			0.061[b]			0.176[b]
Never	504 (79.5%)	130 (20.5%)		402 (63.4%)	232 (36.6%)	
Former	196 (80.3%)	48 (19.7%)		158 (64.8%)	86 (35.2%)	

© 2023 Champion VL et al. *JAMA Network Open.*

	UTD for All cancer screenings			UTD for Any cancer screenings		
	No record of test or outside window (N=769)	Received within 12mo window (N=194)	p value	No record of test or outside window (N=616)	Received within 12mo window (N=347)	p value
Current	51 (89.5%)	6 (10.5%)		42 (73.7%)	15 (26.3%)	
Unknown	18 (64.3%)	10 (35.7%)		14 (50.0%)	14 (50.0%)	
Baseline BMI			0.273[c]			0.363[c]
Mean (SD)	31.5 (7.7)	30.8 (7.1)		31.6 (7.7)	31.0 (7.4)	
Median (Q1, Q3)	30.3 (26.3, 36.4)	29.0 (25.8, 35.4)		30.2 (26.4, 36.3)	29.6 (25.7, 35.8)	
Range	18.5 - 69.3	19.9 - 60.1		18.8 - 69.3	18.5 - 60.1	
N-Miss	268	50		212	106	
Baseline BMI categories			0.017[b]			0.331[b]
Normal	98 (77.8%)	28 (22.2%)		76 (60.3%)	50 (39.7%)	
Overweight	139 (72.8%)	52 (27.2%)		114 (59.7%)	77 (40.3%)	
Obese	264 (80.5%)	64 (19.5%)		214 (65.2%)	114 (34.8%)	
Unknown	268 (84.3%)	50 (15.7%)		212 (66.7%)	106 (33.3%)	
**Prior cancer screening behavior**						
Have you ever had a mammogram?			0.001[a]			0.014[a]
No	32 (100.0%)	0 (0.0%)		27 (84.4%)	5 (15.6%)	
Yes	737 (79.2%)	194 (20.8%)		589 (63.3%)	342 (36.7%)	
Have you ever had a Pap test?			0.589[a]			1.000[a]
No	4 (100.0%)	0 (0.0%)		3 (75.0%)	1 (25.0%)	
Yes	764 (79.7%)	194 (20.3%)		612 (63.9%)	346 (36.1%)	

© 2023 Champion VL et al. *JAMA Network Open.*

	UTD for All cancer screenings			UTD for Any cancer screenings		
	No record of test or outside window (N=769)	Received within 12mo window (N=194)	p value	No record of test or outside window (N=616)	Received within 12mo window (N=347)	p value
N-Miss	1	0		1	0	
Have you ever done a fecal occult blood test or stool blood test or FIT using a home test kit?			0.553[a]			0.098[a]
No	600 (79.4%)	156 (20.6%)		474 (62.7%)	282 (37.3%)	
Yes	164 (81.6%)	37 (18.4%)		139 (69.2%)	62 (30.8%)	
N-Miss	5	1		3	3	
Have you ever had a colonoscopy?			0.053[a]			0.946[a]
No	418 (82.3%)	90 (17.7%)		324 (63.8%)	184 (36.2%)	
Yes	351 (77.1%)	104 (22.9%)		292 (64.2%)	163 (35.8%)	
Have you ever done a FOBT or had a colonoscopy?						
No	344 (82.3%)	74 (17.7%)		261 (62.4%)	157 (37.6%)	
Yes	425 (78.0%)	120 (22.0%)		355 (65.1%)	190 (34.9%)	
Unknown	0 (0%)	0 (0%)		0 (0%)	0 (0%)	
**Have you ever had a mammogram, FOBT, colonoscopy or PAP test?**						
No	1 (100.0%)	0 (0.0%)		1 (100.0%)	0 (0.0%)	
Yes	768 (79.8%)	194 (20.2%)		615 (63.9%)	347 (36.1%)	
Unknown	0 (0%)	0 (0%)		0 (0%)	0 (0%)	
**Health care encounter experiences**						
	UTD for All cancer screenings			UTD for Any cancer screenings		

© 2023 Champion VL et al. *JAMA Network Open.*

	UTD for All cancer screenings			UTD for Any cancer screenings		
	No record of test or outside window (N=769)	Received within 12mo window (N=194)	p value	No record of test or outside window (N=616)	Received within 12mo window (N=347)	p value
Has a doctor or health care provider ever suggested that you have a mammogram?			0.125[a]			0.582[a]
No	30 (90.9%)	3 (9.1%)		23 (69.7%)	10 (30.3%)	
Yes	730 (79.6%)	187 (20.4%)		586 (63.9%)	331 (36.1%)	
N-Miss	9	4		7	6	
Has a doctor ever suggested that you have a Pap test?			0.015[a]			0.034[a]
No	96 (88.9%)	12 (11.1%)		79 (73.1%)	29 (26.9%)	
Yes	662 (78.8%)	178 (21.2%)		527 (62.7%)	313 (37.3%)	
N-Miss	11	4		10	5	
Has a doctor or health care provider ever recommended that you do a stool blood test?			0.727[a]			0.885[a]
No	508 (80.5%)	123 (19.5%)		407 (64.5%)	224 (35.5%)	
Yes	244 (79.5%)	63 (20.5%)		196 (63.8%)	111 (36.2%)	
N-Miss	17	8		13	12	
Has a doctor ever recommended you have a colonoscopy?			0.073[a]			0.581[a]
No	193 (83.9%)	37 (16.1%)		143 (62.2%)	87 (37.8%)	
Yes	558 (78.4%)	154 (21.6%)		458 (64.3%)	254 (35.7%)	
N-Miss	18	3		15	6	
Has a doctor ever recommended you have a FOBT or colonoscopy?			0.248[b]			0.418[b]

© 2023 Champion VL et al. *JAMA Network Open.*

	No record of test or outside window (N=769)	Received within 12mo window (N=194)	p value	No record of test or outside window (N=616)	Received within 12mo window (N=347)	p value
No	160 (83.3%)	32 (16.7%)		116 (60.4%)	76 (39.6%)	
Yes	603 (79.1%)	159 (20.9%)		495 (65.0%)	267 (35.0%)	
Unknown	6 (66.7%)	3 (33.3%)		5 (55.6%)	4 (44.4%)	
Has a doctor ever recommended you have a mammogram, FOBT, colonoscopy or PAP test?			0.055[b]			0.148[b]
No	15 (100.0%)	0 (0.0%)		13 (86.7%)	2 (13.3%)	
Yes	748 (79.7%)	191 (20.3%)		598 (63.7%)	341 (36.3%)	
Unknown	6 (66.7%)	3 (33.3%)		5 (55.6%)	4 (44.4%)	
Have you received any reminders from your health care facility to tell you that it was time to have a mammogram?			0.094[a]			0.780[a]
No	305 (82.7%)	64 (17.3%)		238 (64.5%)	131 (35.5%)	
Yes	433 (78.0%)	122 (22.0%)		352 (63.4%)	203 (36.6%)	
N-Miss	31	8		26	13	
Have you received any reminders from your health care facility to tell you that it was time to have a Pap test?			0.024[a]			0.175[a]
No	539 (81.7%)	121 (18.3%)		429 (65.0%)	231 (35.0%)	
Yes	199 (74.8%)	67 (25.2%)		160 (60.2%)	106 (39.8%)	
N-Miss	31	6		27	10	

© 2023 Champion VL et al. *JAMA Network Open.*

	UTD for All cancer screenings			UTD for Any cancer screenings		
	No record of test or outside window (N=769)	Received within 12mo window (N=194)	p value	No record of test or outside window (N=616)	Received within 12mo window (N=347)	p value
Have you received any reminders from your health care facility to tell you that it was time to have a stool blood test?			1.000[a]			1.000[a]
No	707 (79.7%)	180 (20.3%)		567 (63.9%)	320 (36.1%)	
Yes	43 (79.6%)	11 (20.4%)		35 (64.8%)	19 (35.2%)	
N-Miss	19	3		14	8	
Have you received any reminders from your health care facility to tell you that it was time to have a colonoscopy?			0.017[a]			0.014[a]
No	643 (81.1%)	150 (18.9%)		519 (65.4%)	274 (34.6%)	
Yes	103 (72.0%)	40 (28.0%)		78 (54.5%)	65 (45.5%)	
N-Miss	23	4		19	8	
Have you received any reminders from your health care facility to tell you that it was time to have a FOBT or colonoscopy?			0.047[b]			0.062[b]
No	635 (81.3%)	146 (18.7%)		513 (65.7%)	268 (34.3%)	
Yes	121 (72.9%)	45 (27.1%)		94 (56.6%)	72 (43.4%)	
Unknown	13 (81.2%)	3 (18.8%)		9 (56.2%)	7 (43.8%)	
Have you received any reminders to have a mammogram, FOBT, colonoscopy or PAP test?			0.049[b]			0.487[b]
No	287 (83.9%)	55 (16.1%)		226 (66.1%)	116 (33.9%)	
Yes	474 (77.7%)	136 (22.3%)		384 (63.0%)	226 (37.0%)	

© 2023 Champion VL et al. *JAMA Network Open.*

	UTD for All cancer screenings			UTD for Any cancer screenings		
	No record of test or outside window (N=769)	Received within 12mo window (N=194)	p value	No record of test or outside window (N=616)	Received within 12mo window (N=347)	p value
Unknown	8 (72.7%)	3 (27.3%)		6 (54.5%)	5 (45.5%)	
**Cancer screening contemplation**						
Are you planning to have a mammogram in the next 6 months?			0.004[a]			< 0.001[a]
No	363 (83.3%)	73 (16.7%)		305 (70.0%)	131 (30.0%)	
Yes	335 (75.3%)	110 (24.7%)		262 (58.9%)	183 (41.1%)	
N-Miss	71	11		49	33	
Are you planning to have a Pap test in the next 6 months?			< 0.001[a]			< 0.001[a]
No	531 (82.6%)	112 (17.4%)		436 (67.8%)	207 (32.2%)	
Yes	170 (72.0%)	66 (28.0%)		129 (54.7%)	107 (45.3%)	
N-Miss	68	16		51	33	
Are you planning to have a home stool blood test in the next 6 months?			0.201[a]			0.283[a]
No	592 (80.2%)	146 (19.8%)		479 (64.9%)	259 (35.1%)	
Yes	63 (74.1%)	22 (25.9%)		50 (58.8%)	35 (41.2%)	
N-Miss	114	26		87	53	
Are you planning to have a colonoscopy in the next 6 months?			0.001[a]			0.004[a]
No	589 (81.8%)	131 (18.2%)		479 (66.5%)	241 (33.5%)	
Yes	82 (68.3%)	38 (31.7%)		63 (52.5%)	57 (47.5%)	
N-Miss	98	25		74	49	

© 2023 Champion VL et al. *JAMA Network Open.*

	UTD for All cancer screenings			UTD for Any cancer screenings		
	No record of test or outside window (N=769)	Received within 12mo window (N=194)	p value	No record of test or outside window (N=616)	Received within 12mo window (N=347)	p value
Are you planning to have a FOBT or colonoscopy in the next 6 months?			0.002[b]			0.003[b]
No	578 (82.3%)	124 (17.7%)		471 (67.1%)	231 (32.9%)	
Yes	136 (70.5%)	57 (29.5%)		104 (53.9%)	89 (46.1%)	
Unknown	55 (80.9%)	13 (19.1%)		41 (60.3%)	27 (39.7%)	
Are you planning to have a mammogram, FOBT, colonoscopy or PAP test in the next 6 months?			< 0.001[b]			< 0.001[b]
No	323 (86.1%)	52 (13.9%)		270 (72.0%)	105 (28.0%)	
Yes	433 (75.7%)	139 (24.3%)		336 (58.7%)	236 (41.3%)	
Unknown	13 (81.2%)	3 (18.8%)		10 (62.5%)	6 (37.5%)	
**Cancer screening beliefs and knowledge**						
Perceived barriers to mammography screening score (range: 9-45)			0.095[c]			0.183[c]
Mean (SD)	18.4 (5.3)	17.7 (5.1)		18.1 (5.3)	18.6 (5.2)	
Median (Q1, Q3)	18.0 (14.0, 22.0)	18.0 (13.0, 21.0)		18.0 (14.0, 21.0)	19.0 (14.5, 22.0)	
Range	9.0 - 34.0	9.0 - 31.0		9.0 - 34.0	9.0 - 33.0	
N-Miss	5	3		4	4	
Perceived barriers to Pap screening score (range:8-40)			0.095[c]			0.657[c]
Mean (SD)	15.9 (5.0)	15.3 (4.9)		15.7 (5.0)	15.9 (4.9)	
Median (Q1, Q3)	16.0 (12.0, 19.0)	15.0 (11.0, 19.0)		16.0 (12.0, 19.0)	16.0 (12.0, 20.0)	

© 2023 Champion VL et al. *JAMA Network Open.*

	UTD for All cancer screenings			UTD for Any cancer screenings		
	No record of test or outside window (N=769)	Received within 12mo window (N=194)	p value	No record of test or outside window (N=616)	Received within 12mo window (N=347)	p value
Range	8.0 - 30.0	8.0 - 28.0		8.0 - 30.0	8.0 - 28.0	
N-Miss	7	3		6	4	
Perceived barriers to FOBT screening score (range: 7-35)			0.538[c]			0.012[c]
Mean (SD)	14.7 (4.8)	14.9 (4.9)		14.4 (4.9)	15.3 (4.7)	
Median (Q1, Q3)	15.0 (11.0, 18.0)	15.0 (10.5, 19.0)		14.0 (10.0, 18.0)	16.0 (11.5, 19.0)	
Range	7.0 - 31.0	7.0 - 26.0		7.0 - 31.0	7.0 - 26.0	
N-Miss	6	3		5	4	
Perceived barriers to colonoscopy screening score (range: 10-50)			0.131[c]			0.069[c]
Mean (SD)	22.2 (6.6)	21.4 (6.3)		21.8 (6.6)	22.6 (6.3)	
Median (Q1, Q3)	23.0 (17.0, 27.0)	22.0 (16.0, 26.0)		22.0 (16.0, 27.0)	23.0 (18.0, 27.0)	
Range	10.0 - 41.0	10.0 - 35.0		10.0 - 41.0	10.0 - 39.0	
N-Miss	5	3		4	4	
Perceived barriers 1st principal component			0.209[c]			0.085[c]
Mean (SD)	0.0 (1.0)	-0.1 (1.0)		-0.0 (1.0)	0.1 (1.0)	
Median (Q1, Q3)	0.1 (-0.8, 0.7)	-0.0 (-0.9, 0.7)		-0.0 (-0.9, 0.7)	0.2 (-0.7, 0.8)	
Range	-2.0 - 3.3	-2.0 - 2.4		-2.0 - 3.3	-2.0 - 2.6	
N-Miss	7	3		6	4	
Perceived benefit of screening score (range: 3-15)			0.590[c]			0.310[c]

© 2023 Champion VL et al. *JAMA Network Open.*

	UTD for All cancer screenings			UTD for Any cancer screenings		
	No record of test or outside window (N=769)	Received within 12mo window (N=194)	p value	No record of test or outside window (N=616)	Received within 12mo window (N=347)	p value
Mean (SD)	11.4 (2.6)	11.5 (2.5)		11.5 (2.7)	11.3 (2.5)	
Median (Q1, Q3)	12.0 (10.0, 13.0)	12.0 (10.0, 12.0)		12.0 (10.0, 13.0)	12.0 (10.0, 12.0)	
Range	3.0 - 15.0	3.0 - 15.0		3.0 - 15.0	3.0 - 15.0	
N-Miss	5	3		4	4	
Perceived cancer risk score (range: 3-9)			0.607[c]			0.913[c]
Mean (SD)	5.4 (1.2)	5.5 (1.3)		5.4 (1.2)	5.4 (1.2)	
Median (Q1, Q3)	6.0 (5.0, 6.0)	6.0 (5.0, 6.0)		6.0 (5.0, 6.0)	6.0 (5.0, 6.0)	
Range	3.0 - 9.0	3.0 - 9.0		3.0 - 9.0	3.0 - 9.0	
N-Miss	6	4		5	5	
Perceived cancer screening self-efficacy score (range: 4-20)			< 0.001[c]			0.126[c]
Mean (SD)	17.5 (3.0)	18.4 (2.2)		17.6 (2.9)	17.9 (2.7)	
Median (Q1, Q3)	18.0 (16.0, 20.0)	20.0 (17.0, 20.0)		19.0 (16.0, 20.0)	19.0 (16.0, 20.0)	
Range	4.0 - 20.0	10.0 - 20.0		4.0 - 20.0	4.0 - 20.0	
N-Miss	6	3		5	4	
Breast cancer knowledge score (range: 0-5)			< 0.001[c]			0.245[c]
Mean (SD)	3.6 (1.1)	4.0 (1.0)		3.7 (1.1)	3.8 (1.0)	
Median (Q1, Q3)	4.0 (3.0, 5.0)	4.0 (3.0, 5.0)		4.0 (3.0, 5.0)	4.0 (3.0, 5.0)	
Range	0.0 - 5.0	0.0 - 5.0		0.0 - 5.0	0.0 - 5.0	
	UTD for All cancer screenings			UTD for Any cancer screenings		

© 2023 Champion VL et al. *JAMA Network Open.*

	No record of test or outside window (N=769)	Received within 12mo window (N=194)	p value	No record of test or outside window (N=616)	Received within 12mo window (N=347)	p value
N-Miss	5	3		4	4	
Cervical cancer knowledge score (range: 0-5)			0.002[c]			0.003[c]
Mean (SD)	2.8 (1.3)	3.1 (1.2)		2.7 (1.3)	3.0 (1.2)	
Median (Q1, Q3)	3.0 (2.0, 4.0)	3.0 (2.0, 4.0)		3.0 (2.0, 4.0)	3.0 (2.0, 4.0)	
Range	0.0 - 5.0	0.0 - 5.0		0.0 - 5.0	0.0 - 5.0	
N-Miss	5	3		4	4	
CRC knowledge score (range: 0-5)			0.001[c]			0.027[c]
Mean (SD)	2.2 (1.3)	2.6 (1.4)		2.2 (1.3)	2.4 (1.3)	
Median (Q1, Q3)	2.0 (1.0, 3.0)	3.0 (2.0, 4.0)		2.0 (1.0, 3.0)	2.0 (1.0, 3.0)	
Range	0.0 - 5.0	0.0 - 5.0		0.0 - 5.0	0.0 - 5.0	
N-Miss	5	3		4	4	
Knowledge score 1st principal component			< 0.001[c]			0.008[c]
Mean (SD)	-0.1 (1.0)	0.3 (1.0)		-0.1 (1.0)	0.1 (1.0)	
Median (Q1, Q3)	0.1 (-0.6, 0.7)	0.4 (-0.3, 1.1)		0.1 (-0.6, 0.7)	0.1 (-0.6, 0.8)	
Range	-3.1 - 2.1	-3.1 - 2.1		-3.1 - 2.1	-3.1 - 2.1	
N-Miss	5	3		4	4	

Note: percentages are row percentages; UTD = up to date.
[a]Two-sided Pearson's chi-squared test.
[b]Two-sided Fisher's exact test.
[c]Two-sided independent-groups t-test.
[d]For the 12% of participants whose medical record health care system location was not re-confirmed at 12 months, the location reported at their baseline interview was used to assess 12-month outcomes; only 5 persons had no medical record data or location confirmation at baseline or 12 months, and, among those 5, the 12-month self-report screening data was available and used for 4 persons in all analyses.

© 2023 Champion VL et al. *JAMA Network Open.*

eTable 2. Logistic Regression for All Cancer Screenings at 12 Months Postbaseline (n = 852): Sensitivity analysis for persons aged 66 or greater were coded to be UTD at baseline for cervical cancer screening.

Characteristic	OR[a]	95% CI[a]	p-value
**Study arm[b]**			
Usual care	—	—	
DVD	1.69	0.94, 3.15	0.090
DVD/Navigator	5.63	3.21, 10.3	<0.001
**Baseline screening status, not UTD for:**			
Breast, colorectal and cervical	—	—	
Breast and colorectal	1.58	0.64, 3.94	0.319
Breast and cervical	2.30	0.84, 6.19	0.100
Colorectal and cervical	0.99	0.36, 2.63	0.982
Breast only	14.9	6.45, 36.8	<0.001
Colorectal only	4.59	2.33, 9.79	<0.001
Cervical only	2.85	1.30, 6.56	0.010
**Age**			
50-54	—	—	
55-59	0.94	0.57, 1.52	0.790
60-64	0.85	0.50, 1.41	0.529
65+	0.40	0.22, 0.72	0.003
**Are you planning to have a mammogram, FOBT, colonoscopy or PAP test in the next 6 months?**			
No	—	—	
Yes	1.93	1.29, 2.94	0.002
Perceived barriers 1st principal component	1.23	0.99, 1.53	0.058
Perceived cancer screening self-efficacy score (range: 4-20)	1.12	1.03, 1.22	0.009
National percentile of block group ADI score	0.99	0.98, 1.00	0.040

[a]OR = Odds Ratio, CI = two-sided 95% confidence interval.
[b]Effect of DVD/Navigator vs DVD: OR = 3.34; CI = 2.19, 5.08, p < 0.001.
Note: Re-confirmation of medical record location at 12 months was also adjusted for in this model (confirmed vs not confirmed at 12 months; OR = 4.76; CI = 2.22, 12.5; p < 0.001). UTD = up to date.

© 2023 Champion VL et al. *JAMA Network Open.*

eTable 3. Logistic Regression for Any Cancer Screening at 12 Months Postbaseline (n = 847): Sensitivity analysis for persons aged 66 or greater were coded to be UTD at baseline for cervical cancer screening.

Characteristic	OR[a]	95% CI[a]	p-value
**Study arm[b]**			
Usual care	—	—	
DVD	1.34	0.86, 2.09	0.198
DVD/Navigator	4.11	2.66, 6.46	<0.001
**Baseline screening status, not UTD for:**			
Breast, colorectal and cervical	—	—	
Breast and colorectal	1.18	0.66, 2.10	0.569
Breast and cervical	1.58	0.79, 3.17	0.200
Colorectal and cervical	0.91	0.50, 1.65	0.761
Breast only	1.54	0.78, 3.06	0.218
Colorectal only	0.50	0.30, 0.82	0.007
Cervical only	0.32	0.17, 0.60	<0.001
**Age**			
50-54	—	—	
55-59	1.17	0.77, 1.76	0.464
60-64	1.05	0.67, 1.66	0.821
65+	1.09	0.65, 1.83	0.742
**How would you describe your household's financial situation right now?**			
Has enough money for special things	—	—	
Can pay bills, but little extra money	0.87	0.60, 1.24	0.434
Has to cut back or has difficulty paying bills	0.46	0.25, 0.83	0.012
**Are you currently working for pay?**			
No	—	—	
Yes - part time	1.41	0.89, 2.23	0.149
Yes - full time	1.53	1.02, 2.29	0.039
**Are you planning to have a mammogram, FOBT, colonoscopy or PAP test in the next 6 months?**			
No	—	—	
Yes	1.86	1.33, 2.62	<0.001
Perceived barriers 1st principal component	1.27	1.06, 1.52	0.011
Knowledge score 1st principal component	1.19	1.00, 1.42	0.047
Perceived cancer screening self-efficacy score (range: 4-20)	1.08	1.01, 1.15	0.030
National percentile of block group ADI score	0.99	0.98, 1.00	0.061

[a]OR = Odds Ratio, CI = two-sided 95% confidence interval.
[b]Effect of DVD/Navigator vs DVD: OR = 3.08; 95% CI = 2.16, 4.37, p < 0.001.
Note: confirmation of medical record location was also adjusted for in this model (confirmed vs not confirmed; OR = 4.55; CI = 2.63, 8.33; p < 0.001). UTD = up to date.

© 2023 Champion VL et al. *JAMA Network Open.*

eAppendix. Intervention Cost Methods

The cost of the interventions can be broken down into two sections: DVD only intervention and DVD plus PN intervention. The DVD-only intervention involves costs of the DVDs, personnel time cost for DVD production and distribution, and consultant costs for DVD production.

The DVD/PN intervention contains costs that were involved in the DVD intervention as well as costs of PN time.

All costs were converted to 2022-dollar values.

DVD Costs**:**

Within the cost of the DVD, there were two main costs. The first was related to personnel time and the second was related to payments given to a third part consultant to assist in the production of the DVD.

Personnel Time Costs**:**

The personnel costs associated with the implementation of the DVD intervention was determined by a detailed account of the operational costs of the intervention, excluding costs that were purely attributable to the research. Personnel contributed to the production and distribution of the DVD by assisting in the development, design, CAB meetings to revise, editing, packaging, and printing of the DVD. Average hours worked over the course of the two-year development were found retrospectively after the completion of the study to determine the cost of the DVD. This information along with salary and fringe benefits were used to calculate much of the cost of the DVD intervention. Finally, costs were updated to reflect 2022 dollars.

Consultant Costs**:**

There a consultant was hired to assist with the production of the DVD. The service performed by the consultant was video production, including strategy, production, and post-production services. The total cost of the consultant was $53,000. This cost along with personnel costs consist of the total cost of the DVD intervention. Finally, costs were updated to reflect 2022 dollars.

DVD/PN Costs:

The costs of the DVD/PN intervention were the summation of the DVD costs and the PN costs and were broken down between the costs of the creation and dissemination of the DVD, which was described in the section above, and the costs of the patient navigators.

PN Costs**:**

Time logs were used to track the broad categories of patient navigator time use, which were used to estimate costs for specific PN activities (e.g., arranging transportation). Navigators were assumed to have 10% effort in the first two years of the study. During this time, they prepared the navigation contact forms and the respective codebooks.

© 2023 Champion VL et al. *JAMA Network Open.*

After the completion of the DVD, which finished in year 2 of the study, 100% PN effort was used to contact the participants to implement the DVD/PN intervention in years 3-5. Their base salary and fringe benefits were used to calculate the cost of the PN component of the DVD/PN intervention. Finally, costs were updated to reflect 2022 dollars.

© 2023 Champion VL et al. *JAMA Network Open.*
