# Supplementary material for: Comparative Effectiveness of 2 Interventions to Increase Breast, Cervical, and Colorectal Cancer Screening Among Women in the Rural US: A Randomized Clinical Trial
Source: JAMA Netw Open. 2023 Apr 28;6(4):e2311004. doi: 10.1001/jamanetworkopen.2023.11004 (PMC10148202; doi:10.1001/jamanetworkopen.2023.11004)
Supplement: Supplement 3. — Data Sharing Statement [file jamanetwopen-e2311004-s003.pdf]

Data Sharing Statement

Champion. Comparative Effectiveness of 2 Interventions to Increase Breast, Cervical, and Colorectal Cancer Screening Among Women in the Rural US. *JAMA Netw Open*. Published April 28, 2023. doi:10.1001/jamanetworkopen.2023.11004

Data
Data available: Yes
Data types: Deidentified participant data
How to access data: Persons wanting access to data will need to contact authors.
When available: With publication

Supporting Documents
Document types: None

Additional Information
Who can access the data: Researchers whose proposal is approved.
Types of analyses: This will depend on the purpose of the analysis.
Mechanisms of data availability: After approval for proposal and signed access data agreement.
